# Hsp90-Associated Immunophilin Homolog Cpr7 Is Required for the Mitotic Stability of [URE3] Prion in *Saccharomyces cerevisiae*

**DOI:** 10.1371/journal.pgen.1005567

**Published:** 2015-10-16

**Authors:** Navinder Kumar, Deepika Gaur, Arpit Gupta, Anuradhika Puri, Deepak Sharma

**Affiliations:** Council of Scientific and Industrial Research-Institute of Microbial Technology, Chandigarh, India; The University of Arizona, UNITED STATES

## Abstract

The role of Hsp70 chaperones in yeast prion propagation is well established. Highly conserved Hsp90 chaperones participate in a number of cellular processes, such as client protein maturation, protein degradation, cellular signalling and apoptosis, but little is known about their role in propagation of infectious prion like aggregates. Here, we examine the influence of Hsp90 in the maintenance of yeast prion [URE3] which is a prion form of native protein Ure2, and reveal a previously unknown role of Hsp90 as an important regulator of [URE3] stability. We show that the C-terminal MEEVD pentapeptide motif, but not the client maturation activity of Hsp90, is essential for [URE3] prion stability. In testing deletions of various Hsp90 co-chaperones known to bind this motif, we find the immunophilin homolog Cpr7 is essential for [URE3] propagation. We show that Cpr7 interacts with Ure2 and enhances its fibrillation. The requirement of Cpr7 is specific for [URE3] as its deletion does not antagonize both strong and weak variant of another yeast prion [*PSI* ^*+*^], suggesting a distinct role of the Hsp90 co-chaperone with different yeast prions. Our data show that, similar to the Hsp70 family, the Hsp90 chaperones also influence yeast prion maintenance, and that immunophilins could regulate protein multimerization independently of their activity as peptidyl-prolyl isomerases.

## Author Summary

Studies on yeast prions have provided insight into the role of various cellular factors involved in amyloid based disorders. Among the various cellular components the chaperone network has emerged as a crucial regulator of formation and propagation of yeast prions. The chaperone machinery primarily consists of Hsp70 and Hsp90 families of proteins, whose activity is further modulated by multiple co-chaperones. Though it is known that Hsp70s and its co-factors influence prion strength and stability, the role of Hsp90 chaperone machinery in yeast prion propagation is not clear. Here we examine the role of Hsp90 chaperones and show that the C-terminal MEEVD motif on Hsp90, which is required for interaction with tetratricopeptide repeat domain containing co-chaperones, is crucial for propagation of the yeast prion [URE3]. Further study revealed a novel role of the Hsp90 co-chaperone Cpr7 in the propagation of infectious amyloid and thus broadens our understanding about the cellular role of immunophilins.

## Introduction

Similar to human prion protein (PrP), many yeast proteins have a tendency to undergo conformational conversion into infectious protein particles known as prions [1,2] [3]. The yeast prions have served as a great model system to understand the mechanistic details of prion induction, propagation, formation of prion variants and roles of various cellular factors in prion maintenance [4,5,6]. Two of the most extensively studied yeast prions [*PSI* ^*+*^] and [URE3] are formed of native proteins Sup35 and Ure2 respectively. Sup35 is a translation termination factor and Ure2 is involved in nitrogen catabolism to repress nitrogen uptake from poor nitrogen sources, like proline, when a good nitrogen source such as ammonia, glutamine or asparagine, is present. Though the prion-forming domains of yeast proteins do not show a significant amino acid sequence similarity with many amyloid-forming proteins in mammals, various constituents of cellular machinery that influence yeast prions also affect mammalian amyloid based disorders, suggesting a functional conservation of cellular factors with regard to amyloidosis.

Among various cellular factors, heat shock proteins (Hsps), consisting mainly of the Hsp70 family and its co-chaperones, play a major role in the maintenance of yeast prions [7,8,9,10,11,12]. Hsp104, which acts in coordination with the Hsp70 system, is essential for propagation of yeast prions. Also, Hsp104 overexpression leads to [*PSI* ^*+*^] curing [13,14,15]. When present as the sole source of Ssa Hsp70, Ssa1 antagonizes [URE3], but not [*PSI* ^*+*^], while cells expressing only Ssa2 show a weak [*PSI* ^*+*^] phenotype [16]. The overexpression of the Hsp70 co-chaperone Ydj1 or the Hsp70 nucleotide exchange factor (NEF) Sse1 cure [URE3] by a mechanism that requires interaction with Hsp70 [17,18]. Both Sse1 and Fes1 are required for [URE3], and Sse1 also regulates [*PSI* ^*+*^] induction and propagation in the prion variant specific manner [10,17,19]. Similarly, [*SWI* ^*+*^] another well studied yeast prion is highly sensitive to alteration in activity of Hsp70 and its co-chaperones [20]. It is believed that chaperones, primarily Hsp70s, play crucial roles in promoting fibril growth and replication required for stable prion propagation [21] [22,23] [16]. Though the role of Hsp70 proteins in yeast prion maintenance has been widely studied, not much is known about Hsp90 function in this regard.

The highly conserved Hsp90 family is essential for cellular growth in all eukaryotes. Hsp90 interacts with about 10% of all cellular proteins and the major clients include kinases, growth hormone receptors [24], transcription factors [25] [26] and signal-transduction factors [27] [28]. Each protomer of dimeric Hsp90 consists of three domains: the N-terminal nucleotide binding domain (NBD), middle domain (MD), and a carboxy-terminal domain (CTD). The Hsp90 dimer exists in a dynamic equilibrium between its open and closed conformation. In the open state Hsp90 is dimerized at its C-terminal domain with N-terminal domains separated. The large-scale conformational changes in Hsp90 upon ATP binding result in dimerization of N-terminal ATPase domains trapping substrate in the substrate binding pocket [29,30]. The charged linker region between the amino terminal and middle domains is crucial for dimerization of Hsp90 at the amino-terminal domain [31].

Hsp90 function is regulated by various other proteins that either modulate its ATPase activity or facilitate interaction with its client proteins. Hsp90 co-chaperones can be broadly divided into those with or without tetratricopeptide repeat (TPR) domains. The TPR domain containing co-chaperones such as Sti1, Cpr6, Cpr7, Cns1, Ppt1 and Tah1 compete for binding at the MEEVD motif present at the C-terminus of Hsp90 [32,33,34] and thus regulate different steps of Hsp90 reaction cycle. The deletion of the highly conserved MEEVD pentapeptide does not cause a growth defect in *S*.*cerevisiae* suggesting that even in the absence of the motif, Hsp90 is functional enough to support essential in vivo roles required for cellular viability.

The TPR proteins participate in many cellular processes that include chaperonin activity, phosphatases, transcriptional regulation and cell cycle control [33]. The TPR domain containing Hsp90 co-chaperones influence client protein maturation by regulating Hsp90 ATPase activity, interaction with other cellular partner proteins, and conformational transition among various dynamic states formed during different stages of its reaction cycle. In addition to the TPR domain, Cpr6 and Cpr7 of the cylophilin family also contain a peptidyl-prolyl isomerase (PPIase) domain that catalyzes the cis-trans isomerization of peptide bonds N-terminal to the proline residues in proteins. Deleting the PPIase domain of Cpr7 does not affect yeast growth or Hsp90 activity, and the exact role of its PPIase domain in client maturation remains uncertain [35]. Unlike Cpr6, Cns1, which is essential for yeast cell growth, complements some Cpr7 functions indicating partial functional redundancy among some TPR proteins. Recently, there is emerging interest in the immunophilin family of proteins that includes FK506 binding proteins and cyclophilins for their role in various neurodegenerative diseases [36] [37].

Many Hsp90 clients first enter into the Hsp70-Hsp40 reaction cycle [38]. The bridge proteins Hop/Sti1 form an intermediate complex with Hsp70 and Hsp90 thus facilitating substrate transfer to Hsp90, which remains in an ATP-free open conformation in the complex [39]. The Hsp90 cycle then begins with ATP binding and displacement of Hop/Sti1 with other TPR containing co-chaperones belonging to peptidyl-prolyl-cis/trans isomerase family such as yeast Cpr6 and Cpr7 [34]. Other non-TPR co-chaperones such as p23/Sba1 and Aha1 influence client maturation by modulating Hps90 conformational changes and its ATPase activity [40,41,42].

What determines substrate specificity and how a large pool of substrates gets partitioned between Hsp70 and Hsp90 is poorly understood. In contrast to the known role of cytosolic Hsp70s, not much is known about the role of Hsp90 and its co-chaperones on yeast prion formation and propagation. Though Hsp90 inhibition has no effect on [*PSI* ^*+*^], indirect evidence suggests some unknown role of Hsp90 proteins in yeast prions maintenance. Alterations of heat shock transcription factor, whose activity is regulated by Hsp90, influence de novo [*PSI* ^*+*^] formation and propagation [43]. Similarly, Hsp90 co-chaperones Sti1 and Cpr7 are important for [*PSI* ^*+*^] curing upon Hsp104 overexpression [44,45]. Furthermore, Cpr7 is involved in maintaining conformation of prion variants [46]. Similarly, other TPR co-chaperone such as overexpressed Sgt2, which is involved in the guided entry of tail-anchored proteins (GET) trafficking pathway, enhances excess Ssa Hsp70 mediated protection of [*PSI* ^*+*^] from curing by excess Hsp104 [47].

Here, we explore whether Hsp90 plays any role, direct or indirect, on yeast prion propagation. We show that [URE3] propagation is not affected by inhibition of Hsp90 or by deleting either of the genes encoding Hsc82 or Hsp82. Interestingly, though mutations affecting Hsp90 conformational changes have no effect on [URE3], those lacking the C-terminal pentapeptide motif destabilize [URE3], revealing that protein-protein complexes of Hsp90 and its TPR co-chaperones has a crucial role in prion maintenance. Our data suggest that the decrease in [URE3] stability in cells expressing Hsp90ΔMEEVD is due to loss of Hsp90 interaction with Cpr7. Our study uncovers a role of Cpr7 in [URE3] propagation and that it is unique among known Hsp90 co-chaperones in its being required for prion maintenance. Our data suggest that Hsp90 controls the fate of not only its client proteins but also other cellular substrates through the functional modulation of other members of the chaperone machinery.

## Results

### The conserved C-terminal MEEVD motif of Hsp90 is required for [URE3] stability

**Fig 1:**
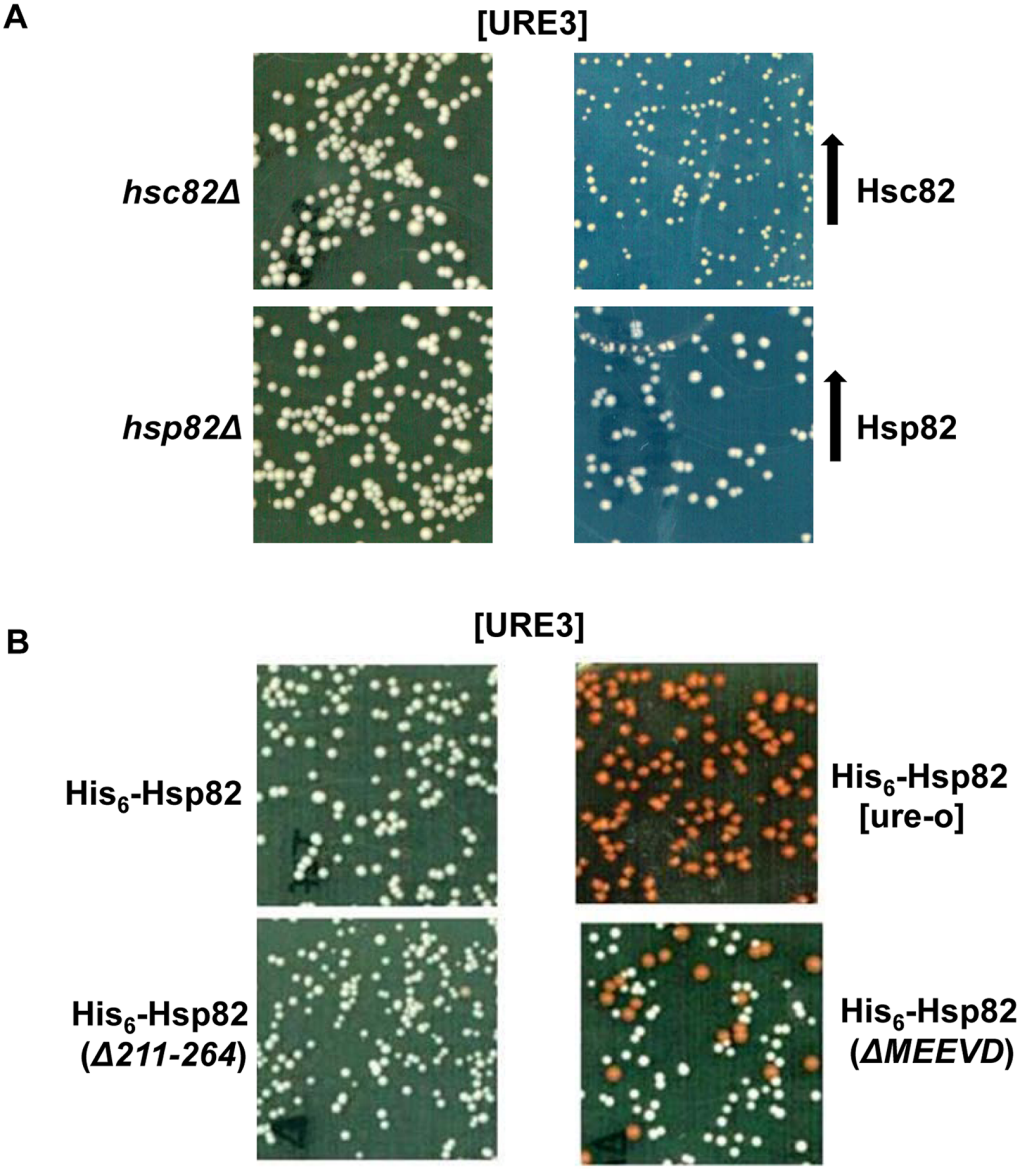
Deleting the MEEVD motif of Hsp90 destabilizes [URE3]. **(A)** Effect of deletion and overexpression of Hsp90 on [URE3]. *Hsp82Δ* (SY295) or *Hsc82Δ* (SY297) cells grown overnight were subcultured into YPAD medium and grown from O.D._600nm_ of 0.02 to 1.7. Cells were then plated onto ½ YPD plates and monitored after 3 days of incubation at 30°C. For overexpression studies, SY187 was transformed with plasmid (pRS426P_GPD_-His_6_-Hsp82 or pRS426P_GPD_-His_6_-Hsc82) encoding His_6_-Hsp82 or His_6_-Hsc82 and plated onto uracil deficient solid SD medium with limiting adenine. As seen by white colony color phenotype, no adverse effect on [URE3] stability was observed upon deletion or overexpression of Hsp90 isoforms. **(B)** The strains constructed as described in Materials and Methods express the indicated Hsp90 isoform as the sole cytosolic Hsp90. Cells were grown from O.D._600nm_ of 0.02 to 1.7 before plating onto ½ YPD medium. As seen by red colony color phenotype the frequency of [ure-o] colonies increases upon deletion of MEEVD motif.

It is known that Hsp70 and Hsp90 family proteins influence folding and maturation of many cellular proteins, yet what determines chaperone specificity is not entirely clear. Whether yeast prions that are well known Hsp70 substrates also require Hsp90 remains unclear. In order to explore the role of Hsp90 chaperones on yeast prions, *S*. *cerevisiae* strains having deletion of either *HSC82* or *HSP82* were constructed. As seen in Fig 1A, no significant effect on [URE3] was observed suggesting that the presence of either Hsp90 is enough to support stable prion propagation. Alternatively, the Hsp90 family of proteins may be dispensable for [URE3] propagation.

To address the latter possibility, we cultured yeast [URE3] cells lacking *HSC82* (strain SY297) in the presence of the known Hsp90 inhibitor 17-AAG (S1 Fig). The inhibitor competes with ATP for binding to Hsp90 and thus inhibits its activity [48]. Inhibition of Hsp90 leads to Hsp70 overexpression and a defect in the maturation of its client proteins [49]. We first examined the effect of 17-AAG on Hsp70 expression and the steady state level of the Hsp90 client v-Src in yeast strain SY136. The SY136 strain was transformed with plasmid (pRS316P_GAL_-FLAG-vSrc) encoding for FLAG-tagged v-Src. The transformants were further cultured in liquid SGal media lacking uracil in the presence and absence of 17-AAG. As expected, incubation with 17-AAG (50μM in DMSO) upregulated Hsp70 expression. Also 17-AAG treated cells showed reduced steady state level of Hsp90 client protein v-Src (S1A Fig) with no significant change in its mRNA levels (S1B Fig). However 17-AAG (100μM in DMSO) mediated inhibition of Hsp90 had no effect on [URE3] stability, suggesting the Hsp90 function crucial for client maturation does not contribute much to [URE3] propagation (S1C Fig).

Hsp90 is essential and thus to examine whether Hsp90 influences [URE3], we monitored prion propagation in yeast cells expressing the previously known Hsp90 mutants, His_6_-Hsp82Δ211–264 or His_6_-Hsp82ΔMEEVD as the sole source of Hsp90 [34]. Amino acids 211–264 form a part of the charged linker region required for Hsp90 conformational changes and thus cells expressing His_6_-Hsp82Δ211–264 as the only Hsp90 grow poorly at 37°C [34]. When combined with *cpr7Δ* the growth defects are additive even at 30°C. Also, strains expressing a similar construct Hsp90Δ211–263 show reduced activation and lower steady state level of its client v-Src [31]. The highly conserved C-terminal pentapeptide MEEVD is a common interaction site where various Hsp90 co-chaperones possessing TPR domains compete for binding to Hsp90. As seen in Fig 1B, a similar frequency (>99%) of [URE3] colonies was observed in cells expressing wt His_6_-Hsp82 or His_6_-Hsp82Δ211–264. However, cells expressing His_6_-Hsp82ΔMEEVD exhibit an increased [ure-o] phenotype as seen by an increase in the number of red colonies (about 25 ± 5%), which arise from cells that lost [URE3]. The increase in frequency of [ure-o] colonies is not due to differences in steady state abundance of Hsp90, Hsp70, Ydj1, or Sse1, as shown in S2 Fig. The results indicate that the presence of the C-terminal MEEVD motif is required for the stability of [URE3]. Overall the data suggest an important previously unknown role of Hsp90, independent of its client maturation activity, in the propagation of prions.

### Cpr7 is required for [URE3] stability

**Fig 2:**
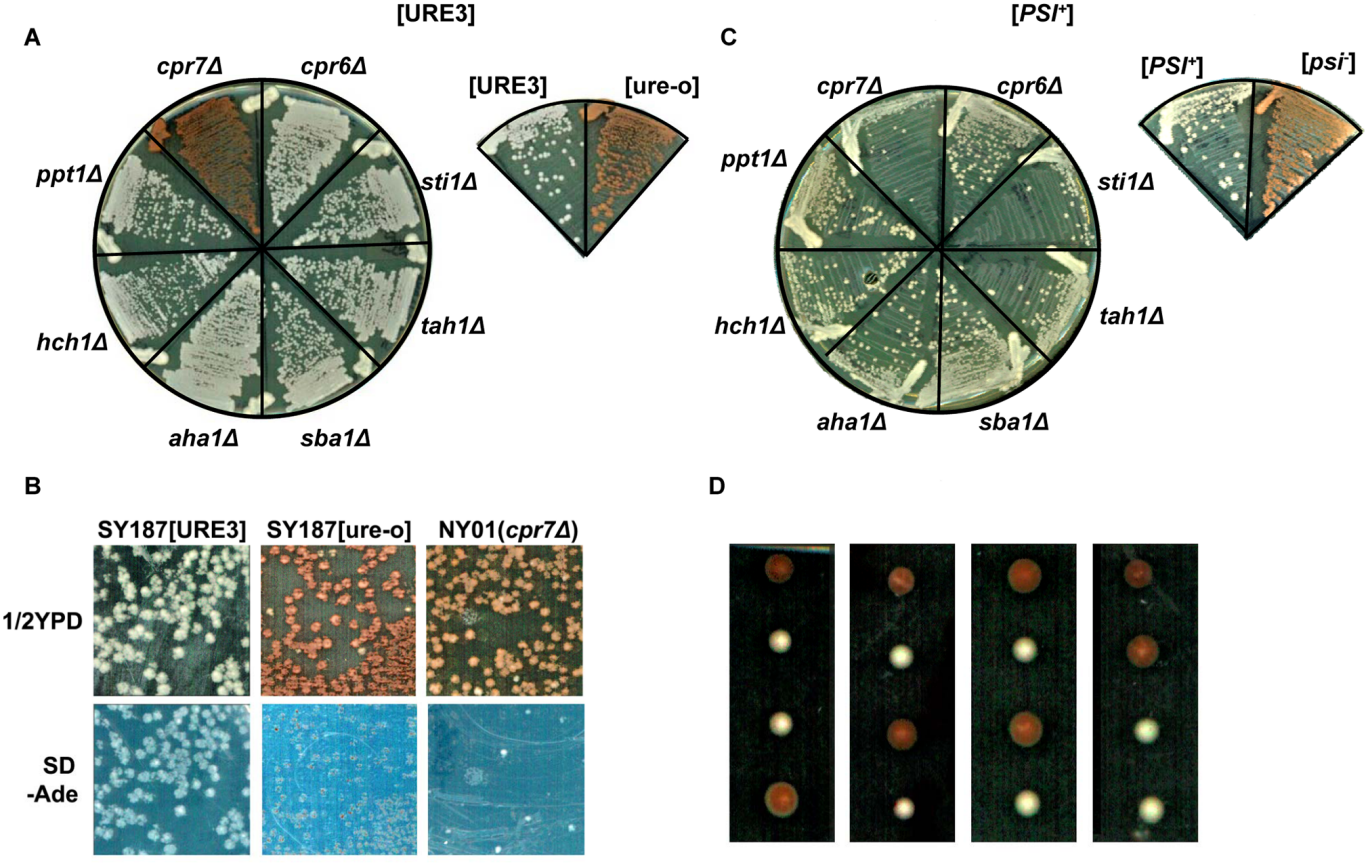
Cpr7 is required for [URE3] stability. (A) Effect of deleting genes encoding Hsp90 co-chaperones on prion propagation. Multiple single-knockout strains constructed as described in Materials and Methods were streaked onto a ½ YPD plate and [URE3] was monitored after 3 days of incubation at 30°C and 2 days at room temperature. As seen, the deletion of gene encoding Cpr7 leads to [ure-o] phenotype. (B) Cells were grown in YPAD liquid medium, further grown from O.D._600nm_ = 0.02 to 1.7 and then plated on ½ YPD. The colonies were then replica-plated onto ½ YPD and adenine deficient plates. As seen, though SY187 [URE3] cells grow normally on adenine deficient medium, only about 2–3% of colonies from *cpr7Δ* strain grew on solid medium lacking adenine. (C) The effect of Hsp90 co-chaperone deletion on [*PSI* ^*+*^] propagation. Deletion strains of the *ade2-1* background were assessed as in panel (A). (D) [URE3] diploid strain heterozygous for *cpr7Δ* (*CPR7*/*cpr7*::*KanMX*) was sporulated and dissected. [URE3] phenotype segregated 2:2 and all *cpr7Δ* (G418 resistant) spores were found to be [ure-o] as seen by red colony color phenotype.

As compromising Hsp90 client maturation activity does not affect [URE3], the appearance of [ure-o] cells in the His_6_-Hsp82ΔMEEVD strain points toward a crucial role of Hsp90 co-chaperones in prion propagation. In order to examine the role of various Hsp90 co-factors, we created many single-knockout *S*. *cerevisiae* strains each lacking an Hsp90 co-chaperone (Sti1, Sba1, Cpr6, Cpr7, Ppt1, Tah1, Hch1 or Aha1) and monitored [URE3]. As shown in Fig 2A the deletion of non-TPR Hsp90 co-chaperones (Sba1, Hch1 or Aha1) had no effect on the prion propagation. Among the TPR domain containing proteins, only deletion of the gene encoding Cpr7 profoundly affects [URE3] stability as seen by the appearance of red colony color as well as poor growth on solid medium lacking adenine (Fig 2A and 2B). To examine whether this effect was [URE3] specific, we further constructed similar single-knockouts in strain 779-6A propagating the other well studied yeast prion [*PSI* ^*+*^]. In contrast to its requirement for [URE3], lack of Cpr7 had no effect on [*PSI* ^*+*^] stability which is also in agreement with a previously reported study [50]. Similarly, other knockout strains carrying a single gene deletion encoding one of seven other Hsp90 co-chaperones also show no apparent differences in [*PSI* ^*+*^] stability (Fig 2C). We also examined the effect of deleting Cpr7 on strong and weak [*PSI* ^*+*^] variants and found no difference in the prion stability in the presence and absence of Cpr7 (S3 Fig). Thus both weak and strong [*PSI* ^*+*^] variants remain largely unaffected by Cpr7 deletion which is distinct from the effect of Hsp70 co-chaperone Sse1 where deletion of Sse1 destabilize weak [*PSI* ^*+*^] but has no effect on strong [*PSI* ^*+*^] prions [51].

As described in Materials and Methods, the red phenotype in [ure-o] cells is due to repression of *ADE2* transcription by Ure2 protein. Any cellular activity that negatively affects *ADE2* transcription or Ade2 protein stability, even independently of Ure2, could also produce similar red colony color phenotype as seen for [ure-o]. Thus, it was possible that [URE3] was still present in *cpr7Δ* cells, but its phenotype was masked by lack of Cpr7. In order to explore these possibilities we transformed [ure-o] colonies from the *cpr7Δ* strain with a plasmid encoding Cpr7 (pRS316P_CPR7_-CPR7). If in *cpr7Δ* cells the red colony color is independent of [URE3], or the [URE3] phenotype is masked, then the transformants would show white phenotype upon plasmid based complementation of *CPR7*. Alternatively if the red phenotype of *cpr7Δ* cells is due to the absence of [URE3], the transformants would continue to show red phenotype as the frequency of spontaneous appearance of [URE3] clones is rare. As shown in S4 Fig, more than 99% of transformants show red colony color phenotype suggesting deletion of gene encoding Cpr7 results in loss of [URE3].

In order to further examine whether [URE3] propagation depends upon the presence of Cpr7, we sporulated a [URE3] diploid strain heterozygous for *cpr7Δ* (*CPR7*/*cpr7*::*KanMX*) and dissected multiple tetrads. In yeast the chromosomally inherited traits follow Mendelian segregation in 2:2 fashion following meiosis however the cytoplasmically inherited infectious yeast prion particles segregate in a non-Mendelian fashion and are transmitted to all four spores. Upon tetrad dissection of a diploid strain heterozygous for *CPR7*, [URE3] segregated in a Mendelian 2:2 fashion, as seen by white colony color (Fig 2D). These results suggest the [ure-o] phenotype is linked to one of the segregating genes. All colonies having red colony color, and none of the white colonies, grew normally on a plate containing G418 showing that all [ure-o] colonies lack gene encoding Cpr7. Together our results clearly indicate that stable [URE3] maintenance requires the presence of Cpr7 and that the absence of [URE3] in *cpr7Δ* cells is not due to synthetic lethality of [URE3] with depletion of Cpr7.

### [URE3] loss is not due to altered expression of other chaperones

**Fig 3:**
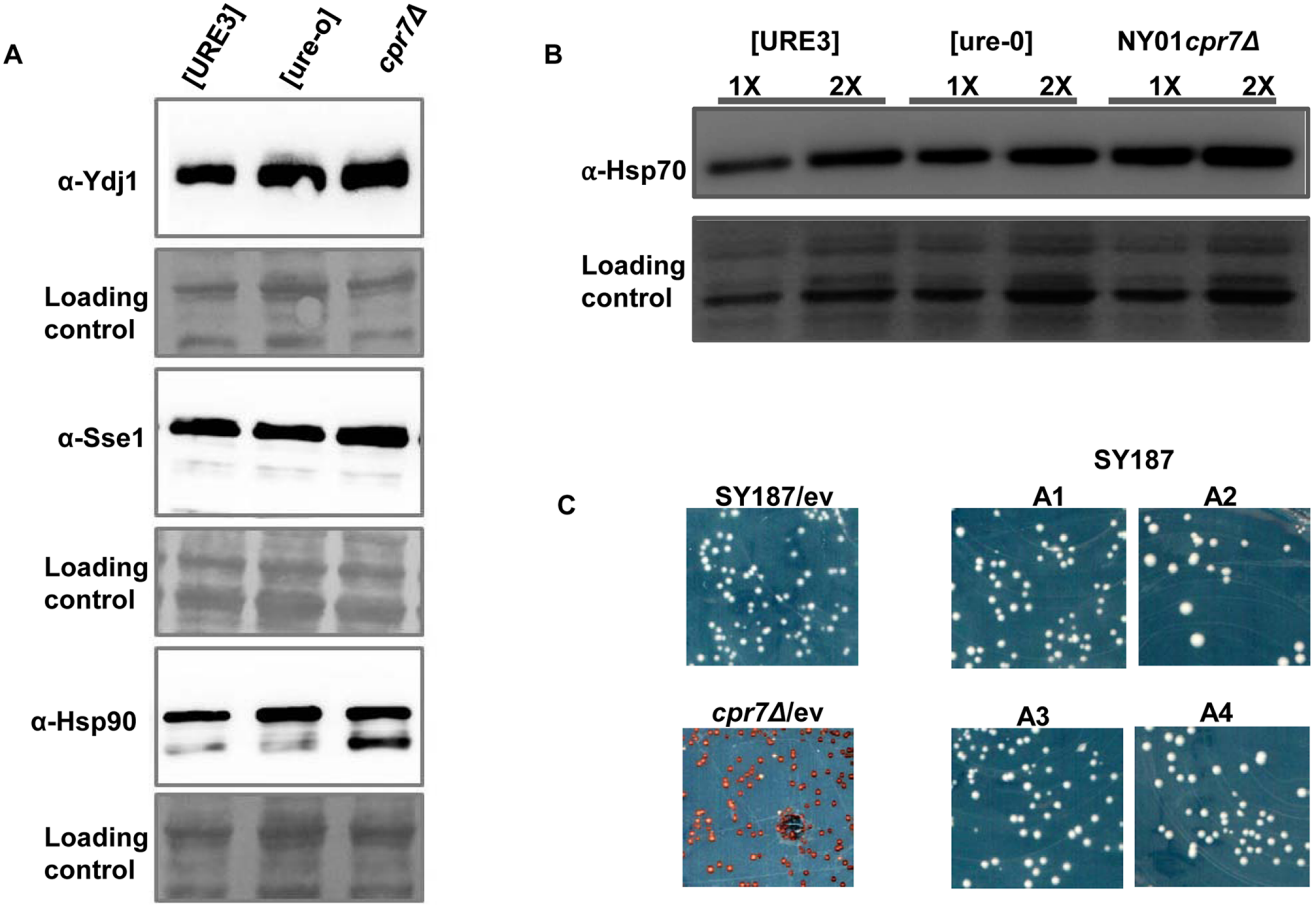
The loss of [URE3] upon Cpr7 deletion is not due to altered expression of other major chaperones. (A) Yeast lysates from wild type (wt) [URE3], wild type [ure-o] and *cpr7Δ* strains were probed with antibodies against Ydj1, Sse1 and Hsp90. The chaperones were found to be expressed at similar levels in the strains examined. Loading control is same blot stained with amido-black. (B) 5μg (1X) or 10μg (2X) of total lysate protein was loaded into each lane and probed with anti Hsp70 antibodies. As seen, increased Hsp70 level was observed in strain lacking Cpr7 as compared to wt [URE3] or wt [ure-o] strains. (C) SY187(wt) expressing additional Ssa Hsp70 from transformed plasmids pRS315P_SSA2_-SSA1/SSA2/SSA3/SSA4 or pRS315 empty vector were spread onto leucine deficient SD solid medium with limiting adenine. As seen by white colony color phenotype of transformants, the increased expression of Ssa Hsp70 supports stable [URE3].

Modulation of Hsp70 activity can alter yeast prion formation and propagation. Similarly, overexpressing the Hsp70 co-chaperones Ydj1 or nucleotide exchange factor Sse1 destabilize [URE3]. In order to explore whether the loss of [URE3] in *cpr7Δ* strain is due to alteration in the overall abundance of Hsp70 and/or its co-chaperones, their relative expression level was monitored in [URE3] and [ure-o] cells of wild type and *cpr7Δ* strains. As seen in Fig 3A, Ydj1, Sse1 and Hsp90 are expressed at similar levels in both wild type and *cpr7Δ* strains, regardless of [URE3] status. In contrast, the Hsp70 level was increased by about 1.5–2.0 fold in the *cpr7Δ* strain as compared to that of wild type strains (Fig 3B).

The four Ssa Hsp70 isoforms in *S*.*cerevisiae* influence [URE3] propagation, and though they are highly homologous they have distinct effects on yeast prions [16]. In order to examine whether the decrease in [URE3] stability in *cpr7Δ* strain is due to an increase in Hsp70, wild type strain SY187 was transformed with a plasmid (pRS315P_SSA2_-SSA1/2/3or4) encoding one of the four Ssa Hsp70 isoforms. Nevertheless, the Leu^+^ primary transformants expressing an additional Ssa Hsp70 isoform show stable [URE3] phenotype similar to that seen for the wild type strain transformed with pRS315 (Fig 3C). These results suggest that increase in Hsp70 upon *CPR7* deletion does not contribute significantly to [URE3] destabilization. Additionally, we previously showed that further subculturing of transformants overexpressing Ssa1 destabilize [URE3] with about 7–8% of cells showing [ure-o] phenotype [52]. However, as the absence of Cpr7 leads to more than 99% of cells showing [ure-o] phenotype, the near complete loss of [URE3] in *cpr7Δ* cells can be interpreted as being caused by something other than an increase in amount of Ssa1 [52].

As a way to confirm that the anti-[URE3] effects of depleting Cpr7 were not due to increased expression of Ssa1, we constructed strain NY17 by deleting the gene encoding Cpr7 in strain NY16 that expresses Ssa2 under Ssa2 promoter as the only source of Ssa Hsp70. As seen by red colony color phenotype and poor growth on Ade^-^ solid growth medium in S5A Fig, depleting Cpr7 caused loss of [URE3]. This loss occurred despite the complete absence of Ssa1, Ssa3 and Ssa4. We examined the abundance of Hsp70 in strains NY16 and NY17 and found it was relatively higher in the *cpr7Δ* strain (S5B Fig). As elevated Ssa2 abundance supports a stable [URE3] phenotype (S5C Fig), the increase in [ure-o] colonies in strain NY17 must be unrelated to an increase in amount of Ssa2.

### The TPR domain of Cpr7 is required for [URE3] propagation

**Fig 4:**
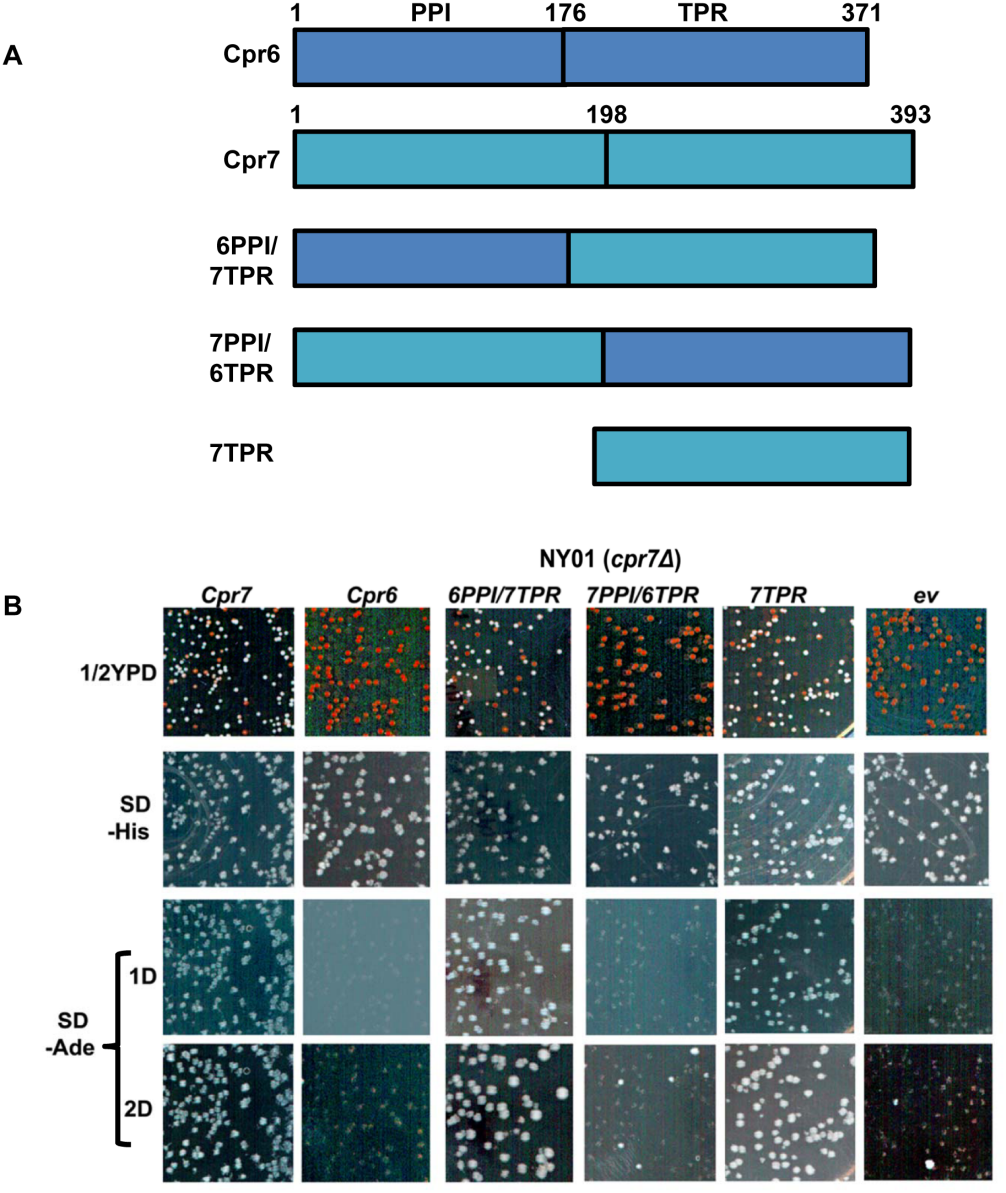
Cpr7 tetratricopeptide domain is important for [URE3] prion propagation. (A) Schematic of domain architecture of Cpr6, Cpr7 and their hybrid proteins. (B) Strains expressing Cpr6, Cpr7 or hybrids in *cpr7Δ* genetic background were constructed and monitored for [URE3] as described in Fig 1A. ½ YPD plates were further replicated onto SD lacking histidine (SD, -His) or adenine (SD, -Ade). Shown is the growth after 1day (1D) or 2 days (2D) of incubation at 30°C after replica plating from ½ YPD plate. As seen by white colony color phenotype cells expressing Cpr7, 6PPI/7TPR or 7TPR support stable [URE3]. Red colony color phenotype is seen only in those colonies that either lost the transformed plasmid (due to growth on ½ YPD), harbour empty plasmid or the plasmid encoding for Cpr6, 7PPI/6TPR.

The Hsp90 associated immunophilins Cpr6 and Cpr7 belong to the PPIase family of enzymes and share more than 50% sequence similarity, yet they can act in distinct cellular processes [53] [54]. In order to examine whether Cpr6 can complement the role of Cpr7 with regard to [URE3], the *cpr7Δ* strain harbouring Cpr7 on a *URA3*-based plasmid (pRS316P_CPR7_-CPR7) was transformed with a plasmid encoding Cpr6 or Cpr7 (pRS413P_TEF_-CPR6 or pRS413P_TEF_-CPR7, respectively) regulated by the TEF promoter. We then used FOA selection to isolate cells having lost the resident *URA3* plasmid. The resulting *cpr7Δ* strains with plasmids encoding Cpr6 or Cpr7 were grown in liquid medium selecting for plasmid maintenance and then spread onto ½ YPD plates. Colony color was noted after incubation at 30°C for 3–4 days, at which time the ½ YPD plates were replicated onto SD medium lacking histidine (for monitoring plasmid loss) or adenine (for monitoring [URE3]). As shown in Fig 4B, cells expressing Cpr7 colonies were white on ½ YPD and grew well on medium lacking adenine, suggesting that the plasmid expressed Cpr7 functionally complements the chromosomal knockout of *CPR7* with regard to [URE3]. In contrast, cells harbouring either an empty plasmid (ev) or a plasmid encoding Cpr6 show similar red colony color on ½ YPD. After continued incubation on adenine deficient growth medium at 30°C, cells expressing Cpr6 grew only poorly, which is similar to cells with the empty plasmid. Thus, Cpr6 was unable to complement Cpr7 function with regard to [URE3] maintenance.

The amino acid identity between the PPIase and TPR domains of Cpr6 and Cpr7 is about 45% and 32% respectively. To examine which domain of Cpr7 is crucial for [URE3] propagation, hybrid proteins, based upon the Cpr6 and Cpr7 as the parent proteins, were constructed in which either the PPIase (6PPI/7TPR) or TPR (7PPI/6TPR) domain of Cpr7 was swapped with that of Cpr6 (Fig 4A) [34] and their ability to support [URE3] was again monitored as described above for Cpr6. As shown in Fig 4B, cells expressing 7PPI/6TPR show red colony color phenotype on ½ YPD medium. The 7PPI/6TPR expressing colonies show either no growth (day 1) or poor growth (day 2) on adenine deficient medium. In contrast, the frequency of white colonies on ½ YPD plate and growth on plates lacking adenine were found to be similar in strains expressing 6PPI/7TPR and wild type Cpr7 suggesting that the TPR domain of Cpr7 is crucial for [URE3] propagation. Furthermore, cells expressing a truncated derivative of Cpr7 lacking the PPIase domain also showed a stable [URE3] phentoype, suggesting peptide-prolyl activity of Cpr7 does not contribute much to the prion stability.

### Cns1 restores [URE3] stability in *cpr7Δ*cells

**Fig 5:**
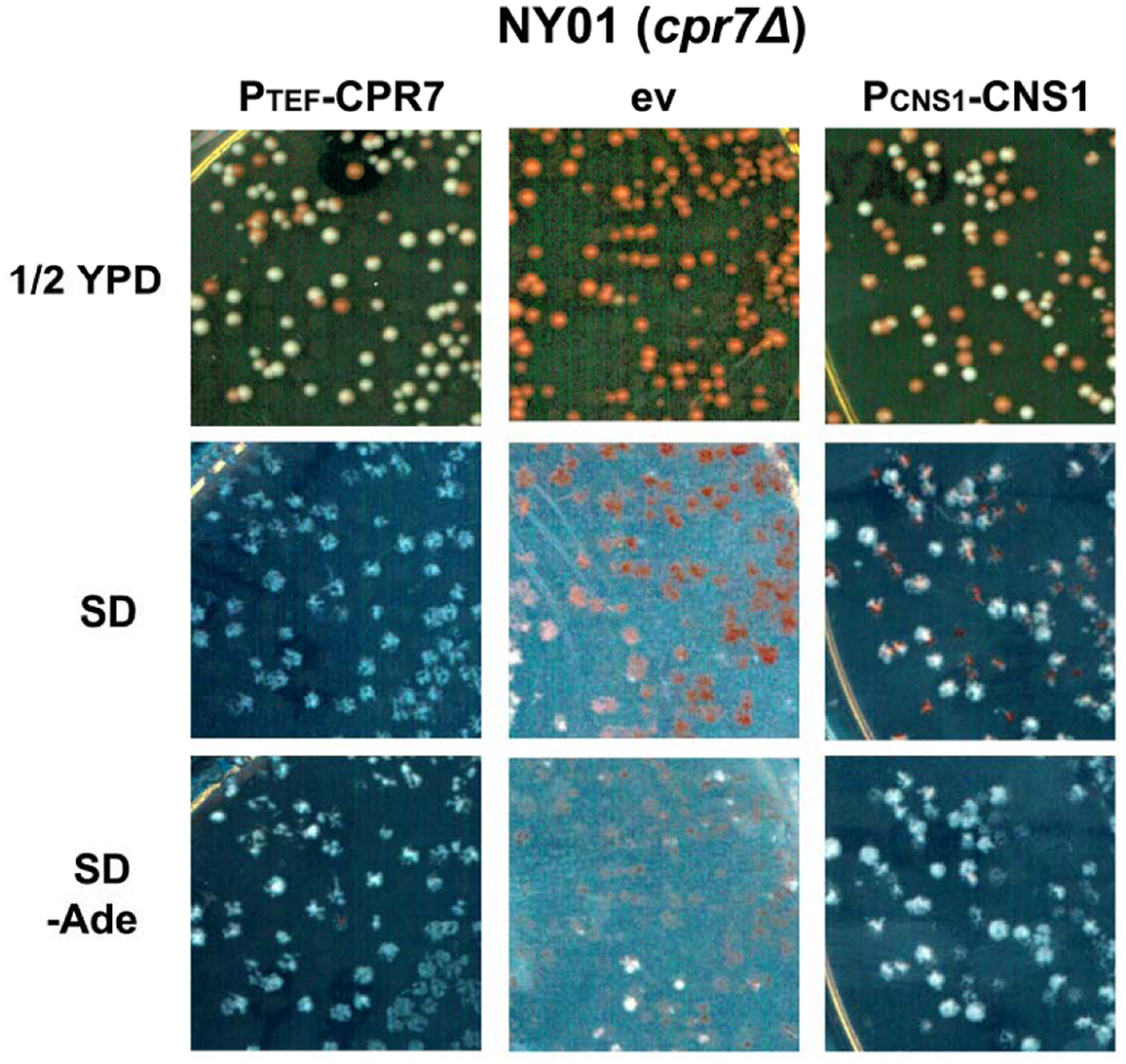
Cns1 complements Cpr7 function required for [URE3] stability. The *cpr7Δ* strain was transformed with pRS426P_CNS1_-CNS1 or pRS413P_TEF_-CPR7, and [URE3] was monitored on ½ YPD plates as described before. All colonies harbouring the Cns1 encoding plasmid show white colony color suggesting functional redundancy of Cns1 and Cpr7 with regard to [URE3] propagation.

Cns1 is unique among the known TPR containing Hsp90 co-chaperones in being essential for yeast cell growth. The 385 amino acid protein contains three TPR motifs and is a suppressor of the growth defect caused by *cpr7Δ*, which suggests partial functional redundancy between Cpr7 and Cns1 [55]. In order to explore whether Cns1 complements for the loss of Cpr7 with regard to [URE3], a plasmid encoding Cns1 (pRS426P_CNS1_-CNS1) was transformed into *cpr7Δ* cells pooled from Ade^-^ medium and transformants were further monitored for [URE3] propagation as described above for Cpr6. As seen before, cells expressing Cpr7 from the constitutive *TEF* promoter showed stable [URE3] on ½YPD (Fig 5). As expected, cells that lost the Cpr7-encoding plasmid (no growth on histidine deficient medium) show red colony color and no growth on Ade^-^ medium. As we saw for Cpr7, we found that cells that express Cns1 are white and grow normally on plates lacking adenine, suggesting that Cns1 functionally complements Cpr7 with regard to [URE3] propagation.

### Cpr7 interacts physically with Ure2

**Fig 6:**
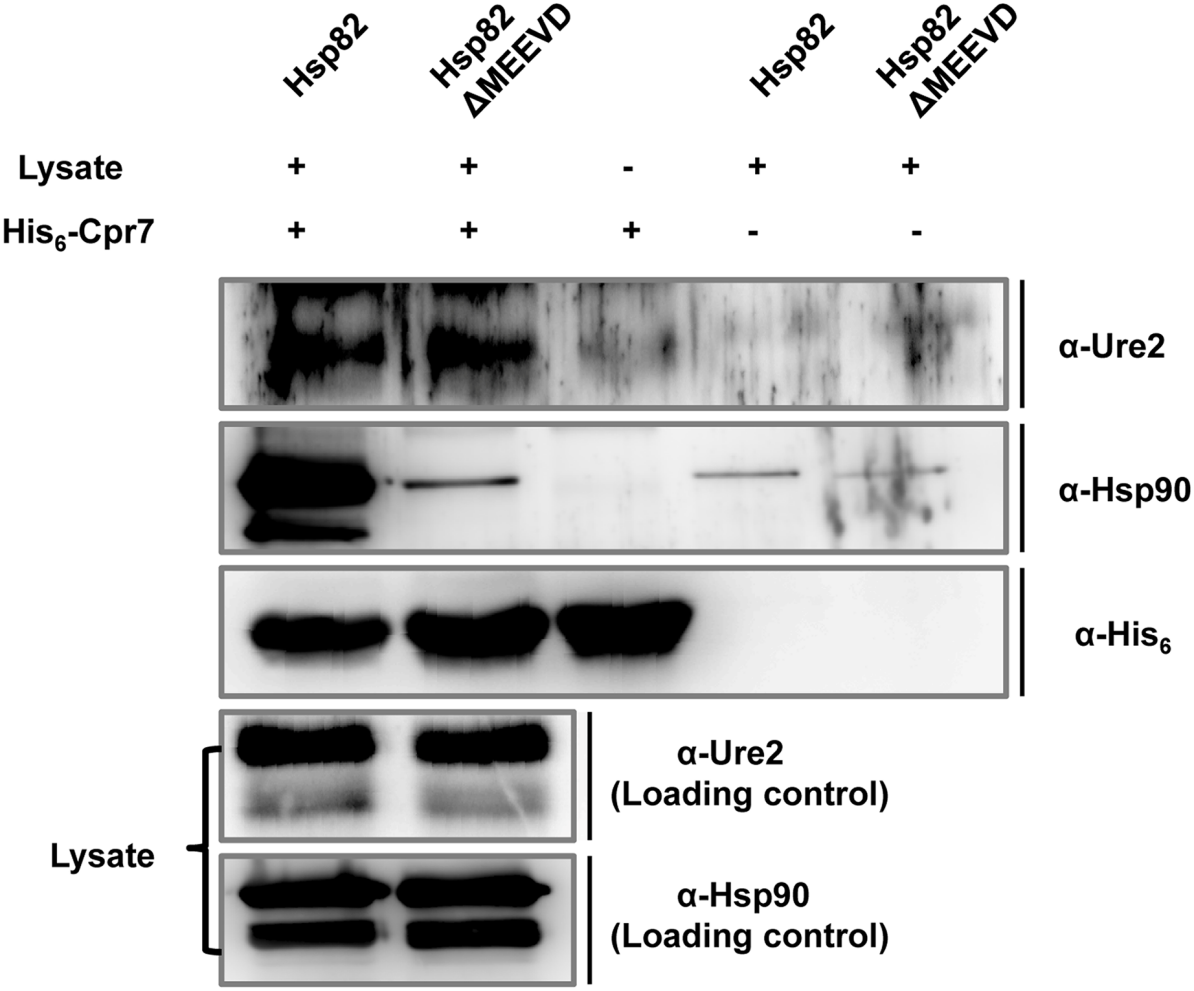
Cpr7 interacts with Ure2. The purified His_6_-Cpr7 was adsorbed onto Cobalt metal affinity resin and further incubated with yeast lysate expressing either Hsp82 or Hsp82ΔMEEVD as sole Hsp90. Upon washing, the fraction bound to His_6_-Cpr7 was eluted with 25mM EDTA, and analyzed for Ure2 and Hsp90 using anti-Ure2 and anti-Hsp90 antibodies, respectively. His_6_-Cpr7 was probed with anti-His_6_ antibody.

Cpr7 belongs to the family of immunophilins that are also known to interact with many unfolded substrates to prevent their aggregation and keep them in a folding competent state [54] [56]. We used a pull-down assay using purified His_6_-Cpr7 as bait to examine interaction of Cpr7 with Ure2. The cell lysate from wild type [ure-o] cells was fractionated and the supernatant was incubated with Cpr7-bound cobalt based metal affinity resin. The unbound proteins were washed and the bound fraction was probed by immunobloting with antibodies specific against Ure2 or Hsp90. In agreement with previous studies, Hsp90 was identified in fractions bound to His_6_-Cpr7 (Fig 6). The deletion of MEEVD leads to a significant decrease in Hsp82 interaction with Cpr7. Interestingly, Ure2 was also detected in the fraction that bound to His_6_-Cpr7. The presence of Ure2 in this fraction suggests that Ure2 either interacts with Cpr7 or Hsp82. We thus examined Ure2 interaction with Cpr7 using cell lysate from cells expressing Hsp82ΔMEEVD. If the presence of Ure2 in the fraction bound to Cpr7 is indirectly due to its interaction with Hsp82, the loss of Cpr7-Hsp82 interaction upon deletion of C-terminus MEEVD motif of Hsp82 would lead to a decrease in Ure2 level in the bound fraction. As shown, however, a similar level of Ure2 was detected in the bound fraction obtained using lysates from cells expressing either Hsp82 or Hsp82ΔMEEVD, suggesting that Cpr7 interacts directly with Ure2.

To substantiate this conclusion, we also examined Cpr7 interaction with Ure2 in vivo by using cells expressing His_6_-Cpr7. We co-transformed strain SY187 with plasmids expressing His_6_-Cpr7 (pRS413P_TEF_-His_6_-CPR7) and Ure2-GFP (pRS426P_GPD_-URE2-GFP) or GFP (pRS426P_GPD_-GFP). Transformants were grown further and the lysate was passed over cobalt based metal affinity resin. As shown in S6 Fig, Ure2 was detected in the bound fraction obtained only from the strain co-expressing His_6_-Cpr7, again suggesting Cpr7 interacts with Ure2 in vivo. Overall, these results indicate that the role of Cpr7 in [URE3] maintenance could be mediated by its ability to interact directly with Ure2.

### Cpr7 enhances Ure2 fibrillation

**Fig 7:**
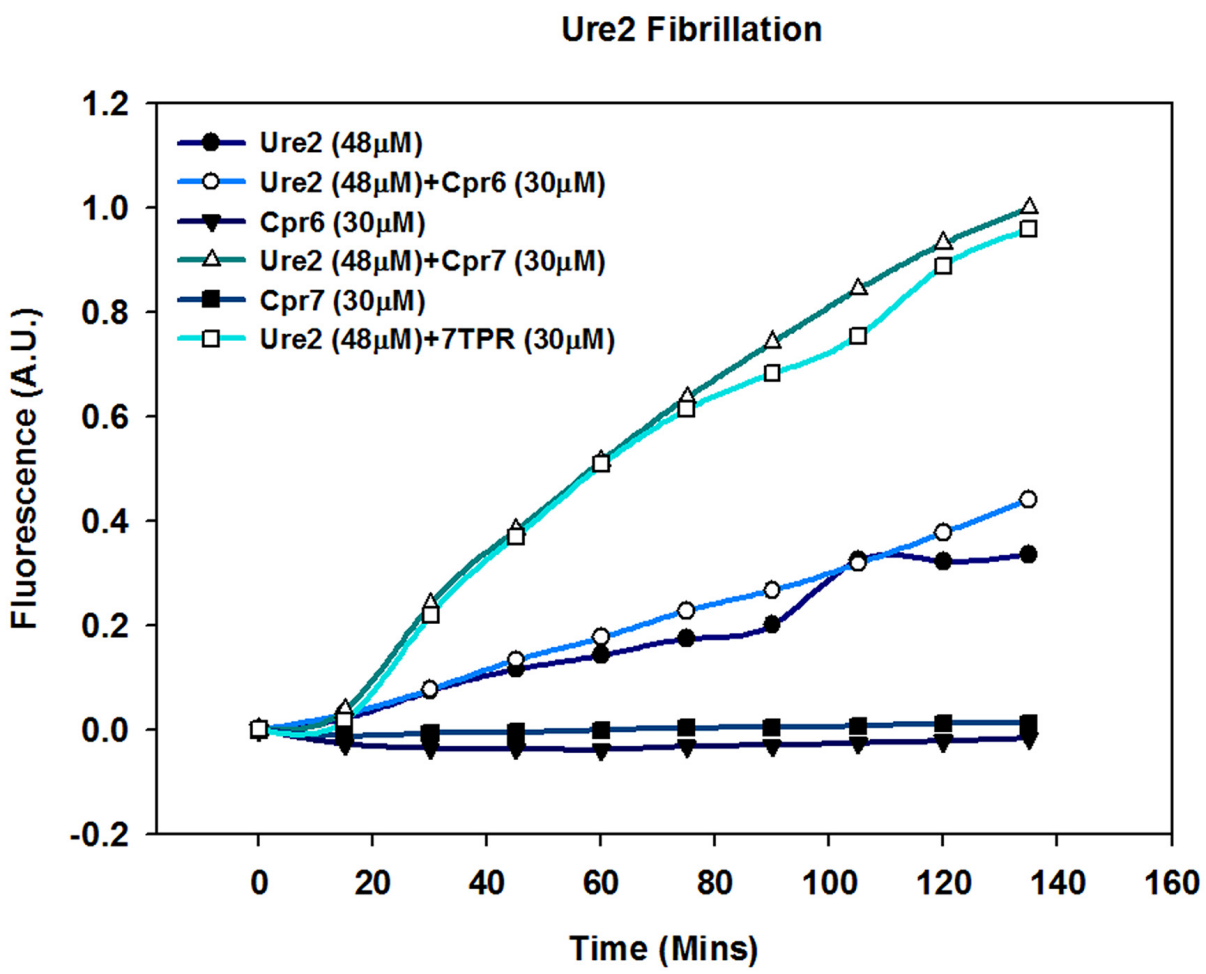
Cpr7 enhances in vitro fibrillization of Ure2. Purified Ure2 (48 μM) was incubated at 37°C with and without Cpr6, Cpr7 or 7TPR (30 μM each) and 500μM ThioflavinT. The ThT fluorescence was monitored at 485nm upon excitation at 450nm. As seen, ThT fluorescence intensity was further enhanced upon incubation of Ure2 with Cpr7 or 7TPR.

As Cpr7 interacts with Ure2 and is required for stable [URE3] propagation, we speculated that Cpr7 might have the ability to modulate Ure2 fibrillation required for stable [URE3] maintenance. In order to examine the effect of Cpr7 on amyloid forming tendency of Ure2, we monitored fibrillation of Ure2 in vitro using Thioflavin T (ThT), a dye that forms fluorescent complexes with amyloids, in the presence and absence of Cpr7. As expected ThT fluorescence intensity increases upon incubation with Ure2 at 37°C, suggesting spontaneous fibrillation of Ure2 with a lag of about 2–3 min. Cpr7 and Cpr6 alone did not affect ThT fluorescence intensity. Pre-incubation of Ure2 with Cpr7 did not affect the lag time. However, the rate of amyloid formation and amyloid yield increased significantly, indicating that Cpr7 promotes Ure2 fibrillation. The increase in Ure2 fibrillation was observed only upon incubation with Cpr7 and not its near homolog Cpr6 (Fig 7). The increase in fluorescence intensity was not due to a direct effect of Cpr7 on ThT as fluorescence intensity was similar when similar amounts of pre-aggregated Ure2 protein, with and without Cpr7, was measured (S7A and S7B Fig).

As the isolated TPR domain of Cpr7 supports [URE3], we also tested if this domain influenced ure2 amyloid formation. Interestingly, as seen for Cpr7, the addition of 7TPR to Ure2 similarly enhanced its fibrillation. These results are in agreement with the in vivo results that showed that 7TPR, but not Cpr6, complemented Cpr7 function with regard to [URE3] propagation. Together these results support the conclusion that Cpr7 plays an important role in [URE3] propagation by enhancing Ure2 fibrillation.

## Discussion

Studies on yeast prions have formed the basis of understanding mechanistic aspects of the formation and propagation of not only of prions, but also other amyloid based disorders. The insight obtained has broadened our understanding not only of the role of various cellular factors in prion maintenance, but also of basic cellular biology. Though both Hsp70 and Hsp90 families of proteins are central to protein quality control in the cell, most of the cellular factors that influence prion propagation belong to the Hsp70 family and its co-chaperones. Whether prion propagation also requires Hsp90 machinery is not yet clear and the role of Hsp90 and its co-chaperones has been shown only to maintain prion conformation and the curing of [*PSI* ^*+*^] upon overexpression of Hsp104 [44] [45] [46,50]. In the present study we show that Hsp90, by virtue of its ability to interact with TPR proteins, is critical for yeast prion propagation. Furthermore we show that Cpr7 is a novel cellular factor required for [URE3] maintenance.

Here we investigated the role of Hsp90 and its co-chaperones on [URE3] propagation. The knockout of genes encoding either Hsc82 or Hsp82 has no adverse effect on [URE3]. This is in contrast to the role of Ssa Hsp70, as different Ssa Hsp70 isoforms show distinct effects on [URE3] [16]. Also, the inhibition of Hsp90 ATPase activity with 17-AAG did not alter [URE3] propagation. Together these results suggest that Hsp90 function specific for the maturation of its client proteins is not crucial for [URE3] maintenance. This is also in agreement with previous observations suggesting that Hsp90 is a more specialized chaperone than Hsp70, which acts on a much broader range of substrates in the cell. The Hsp90 mutant lacking charged linker region (amino acids from 211 to about 263) is defective in ATP dependent amino terminal dimerization, shows a lower steady state level of its client v-Src, and subsequent maturation of its client proteins such as glucocorticoid receptor and v-Src [31]. The strain expressing Hsp90Δ211–264 as sole Hsp90 source also supports a stable [URE3] propagation which provides further evidence that [URE3] stability does not require optimal Hsp90 activity.

Hsp90ΔMEEVD, however, which lacks the binding motif for TPR containing co-chaperones, is incapable of propagating [URE3] stably when it is expressed as the only source of Hsp90. It is possible that Hsp90 interaction with its TPR co-chaperones might modulate the latters' functions in a way that is required for maintenance of [URE3]. Alternatively, the loss of Hsp90 interaction might increase affinity of TPR co-chaperones of Hsp90 toward other cellular proteins having a binding motif similar to MEEVD and such new interactions might lead to [URE3] stability. Collectively the data suggest that although the Hsp90 chaperone cycle including client binding and release may not be involved in [URE3] maintenance, the chaperone might influence [URE3] propagation indirectly through the interaction of its C-terminus with partner co-chaperones that can influence [URE3].

The results from multiple single knockout strains of Hsp90 co-chaperones reveal that Cpr7 is unique among various Hsp90 co-chaperones with regard to its role in the maintenance of mitotically stable [URE3] variant in our yeast strain. Similar to many of the other yeast prions, variants of [URE3] also occur in *S*. *cerevisiae*, and whether or not the essential role of Cpr7 in [URE3] stability is extendable to the other [URE3] variants remains to be explored.

Among the various Hsp90 co-chaperones that facilitate different steps of the Hsp90 reaction cycle, only Cpr7 deletion affects [URE3], suggesting a specific and not a general function of Hsp90 could be essential for [URE3] stability. This is also supported by the observation that cells expressing Hsp82ΔMEEVD and not Hsp82Δ211–264 show [URE3] loss, which points toward a specific role of the C-terminus of Hsp90 in [URE3] propagation. Cpr7 deletion also alters nucleotide dependent Cpr6 interaction with Hsp90, however, as Hsp82Δ211–264, which also leads to a similar effect, does not destabilize [URE3], the alteration of Hsp90-Cpr6 interaction could not be the cause of [URE3] loss from *cpr7Δ* cells [34].

Collectively the results suggest that Cpr7 influences [URE3] propagation directly by participating in processes required for efficient [URE3] propagation, rather than indirectly by influencing Hsp90 function. The possibility that Cpr7 impacts [URE3] directly is in agreement with previous reports showing that cyclophilins could interact with substrate directly to assist their folding and prevent aggregation. Similar to the role of Cpr7 in [URE3] propagation, another related immunophilin FKBP52, present in brain, induces aggregation of the pathological mutant of Tau, Tau-P301L [36,57], suggesting that the role of Cpr7 in [URE3] is not an isolated example of the significance of immunophilins in amyloidosis, but is widespread in higher eukaryotes including mammals.

Yeast cells express several TPR domain containing proteins, but how these interact with their specific partner proteins and influence a specific function of the interacting partner is not clearly understood. Though Cpr7 is primarily an Hsp90 co-chaperone, previous studies suggests that Cpr7 also interacts with Hsp70 [50,58]. Similarly Cpr6 is also known to interact with Hsp70 proteins. Similar to other Hsp90 TPR co-chaperones, such interaction could be mediated by the TPR domain which is known to interact with EEVD-like motifs. However in contrast to the known influence of several other TPR co-chaperones on Hsp70 activity or function, the significance of Cpr6 or Cpr7 interaction on wild type Ssa Hsp70 activity has still not been clearly shown [59]. Indeed, while Cns1 stimulates Ssa1 ATPase activity by about 8-fold, no stimulation was seen by Cpr7 [60]. Similarly, another Hsp90 co-chaperone Sti1 that interacts with Hsp70 strongly stimulates Ssa Hsp70 ATPase activity and helps in the substrate transfer from Hsp70 to Hsp90 [61]. Since our study shows that Cns1 complements Cpr7 function with regard to [URE3], and Cns1, but not Cpr7, stimulates Hsp70 activity, it is more likely that such a profound effect of *cpr7Δ* on [URE3] stability is not mediated entirely through Hsp70. This idea is further supported by the fact the Cpr7 influences Ure2 fibrillation in the absence of any additional cellular factor.

Among the various knockout strains, although *cpr7Δ* decreased [URE3] stability significantly, the lack of effect by its nearly homologous cyclophilin Cpr6 suggests a clear distinction between in vivo roles of these closely related Hsp90 associated cyclophilins. This is also in agreement with previous reports showing non-redundancy in Cpr6 and Cpr7 functions such as their ability to support yeast growth [53]. By using Cpr6 and Cpr7-based hybrid proteins, we find that the TPR domain acts as the regulatory domain to govern functional distinctions between Cpr7 and Cpr6 with regard to [URE3]. As the PPIase domain of Cpr7 is dispensable for [URE3], and a hybrid protein containing the TPR domain from Cpr7 supports stable [URE3] propagation, the distinct action of the two Hsp90 co-chaperones could be due to their different chaperone activity independent of the PPIase function. Stable prion propagation requires continuous growth and breakage of fibrils to generate more seeds for further fibril growth and transmittance to daughter cells. Previous studies show that Cpr7 has more potent chaperone activity than Cpr6, thus it is possible that Cpr7 might be more efficient to help break larger fibrils into smaller ones and hence able to generate more seeds required for [URE3] propagation.

Another TPR co-chaperone, Cns1 restores some Cpr7-dependent activities such as the effect on cell growth, negative regulation of heat shock factors (HSF) and maturation of glucocorticoid receptor [55,62]. Our study now reveals that overexpressed Cns1 also complements Cpr7 function for [URE3] propagation. The HSF and glucocorticoid receptor require Hsp90 and thus Cpr7 deletion could affect these substrates through modulation of the Hsp90 reaction cycle. It is known that Cns1 restores altered Cpr6-Hsp90 interaction upon loss of Cpr7 [34], however, as discussed above, the complementation of Cpr7 function by Cns1 for [URE3] is not due to restoration of Cpr6 interaction with Hsp90, but to redundancy in other Cpr7 functions required for [URE3] stability. As indicated above, Hsp90 client binding function is not crucial for [URE3] stability and the effect of Cpr7 deletion on [URE3] was not caused by altering Hsp90 interaction with Cpr6. Also, we did not find a direct interaction of Hsp90 with Ure2, but did see a direct influence of Cpr7 on Ure2 fibrillation in the absence of Hsp90. Collectively, our results suggest that it is most likely that the effect of Cpr7 deletion on [URE3] is independent of Hsp90 and due to a loss of specific function of Cpr7 in *cpr7Δ* strains. Together our data further suggest that Cns1 could also perform functions independent of Hsp90, and that functional redundancy between Cns1 and Cpr7 is not only limited to their roles as Hsp90 co-chaperones.

In vitro, Cns1 is unable to prevent aggregation of citrate synthase, and also fails to promote refolding of unfolded RNase T1, suggesting that unlike Cpr7, Cns1 not only lacks PPIase activity but also chaperone function for some Cpr7 substrates [60] [54]. Though Cns1 fails to chaperone RNase T1 or Citrate Synthase, it might still be able to influence Ure2 aggregation. As Cns1 lacks PPIase activity, the restoration of [URE3] propagation upon Cns1 overexpression in *cpr7Δ* strain could be through its TPR domain. Though the TPR domain of Cpr7 is only about 10% identical with that of Cns1, the functional complementation of Cpr7 by Cns1 shown in previous and present studies suggests that despite low identity, the structural region critical for Cpr7 function remains partially conserved between the two TPR proteins.

The pull down assay using Cpr7 as a bait protein reveals a novel interaction of Cpr7 with Ure2. Our in vitro ThT assay shows that in the absence of other additional cellular factors, Cpr7 is able to promote Ure2 fibrillation. Though the mechanism by which Cpr7 promotes Ure2 fibrillation is not clear, it is possible that similar to Hsp70s, Cpr7 may act as a chaperone to promote solubilisation of preformed amyloid and during the process breaks a larger fibril into smaller fibrils and thus generates more seeds for further fibrillation. Cpr7 thus might be essential for the prion replication required for stable transmittance of [URE3] prion seeds into daughter cells. Alternatively, in the absence of any additional factor Ure2 assembly could be partitioned between on-pathway and off-pathway intermediates, and the presence of Cpr7 might shift the process more towards the formation of on-pathway intermediates for amyloid formation. Also, as cells expressing the Hsp90ΔMEEVD mutant show [URE3] loss, it is possible that Hsp90 interaction with Cpr7 might modulate its chaperone activity either through inducing conformational changes in Cpr7 or yet other unknown mechanism required for [URE3] maintenance. Hsp90 is previously known to induce conformational changes of its co-chaperone Ppp5 that further activates its phosphatase activity [63]. Alternatively, the retargeting of Hsp90 TPR co-chaperones in the Hsp90ΔMEEVD strain to other cellular factors possessing motifs similar to MEEVD might influence [URE3] stability. Thus, intact Hsp90 could be supporting [URE3] stability by sequestering Cpr7 from binding to its alternative partner protein that in turn might destabilize the prion.

Our data reveal a novel role of cyclophilin Cpr7 in yeast prion [URE3] propagation. Thus, in addition to Hsp70 and its co-factors, Hsp90 and some of its co-chaperones also modulate the maintenance of this yeast prion. The role of immunophilins in amyloid based human diseases such as Alzheimer’s diseases, is emerging, and thus our finding that yeast immunophilins also modulate a yeast prion in a manner required for its efficient propagation provides a genetically tractable system for further understanding the role of immunophilins in amyloidosis. Why [*PSI* ^*+*^] does not require TPR co-chaperone Cpr7 remains to be investigated. Interestingly, cellular factors required for stable [URE3] maintenance, such as Ssa2, are not essential for [*PSI* ^*+*^]. Similarly, Hsp104 overexpression efficiently cures [*PSI* ^*+*^] but not [URE3]. Though more is needed to be explored, it is tempting to speculate that the distinct chaperone requirement for [URE3] and [*PSI* ^*+*^] could be due a specific requirement of Hsp90 chaperone machinery in [URE3] stability.

## Materials and Methods

### Strains and plasmids

Strains and plasmids used are described in Tables 1 and 2, respectively. The wild type strain SY187 (*MATa*, *kar1-1*, *P*_*DAL5*_::*ADE2*, *his3Δ202*, *leu2Δ1*, *trp1Δ63*, *ura3-52*) encodes *ADE2* gene regulated by the *DAL5* promoter for monitoring [URE3]. The *cpr6Δ* (NY14) and *cpr7Δ* strains (NY01) were constructed by genomic integration of *cpr6*::*KanMX* or *cpr7*::*KanMX* cassette, respectively, into SY187 using homologous recombination. The *hsp82Δ* (SY295) and *hsc82Δ* (SY297) strains were constructed using strain MR195 or MR194 (Kind gift from Dr. Masison’s lab), respectively, using standard yeast genetic manipulations. Strain NY02 containing double deletion of *HSC82* and *HSP82* and harbouring plasmid pRS316P_HSP82_-HSP82 was constructed from SY295 using standard yeast genetic methods. The strains NY03, NY04, NY05, NY06 and NY07 were made by shuffling pRS316P_HSP82_-HSP82 in strain NY02 with pRS413P_TEF_-His_6_-HSP82, pRS413P_TEF_-His_6_-HSP82(*ΔMEEVD*), pRS413P_TEF_-His_6_-HSP82(*Δ211–264*), pRS413P_TEF_-HSP82 and pRS413P_TEF_-HSP82(*ΔMEEVD*), respectively. The knockout strain for other Hsp90 co-chaperones (Sti1, Sba1, Cpr6, Cpr7, Ppt1, Tah1, Hch1 or Aha1) was constructed as described above for the *cpr7Δ* strain using standard genetic techniques.

**Table 1:** 

Strains	Genotype	Reference
SY187	*MATa*, *kar 1–1*, *SUQ5*, *P*_*DAL5*_::*ADE2*, *his3Δ202*, *leu2Δ1*, *trp1Δ63*, *ura3-52*, [URE3]	Sharma *et al*, 2011
NY01	*MATa*, *kar 1–1*, *SUQ5*, *P*_*DAL5*_::*ADE2*, *his3Δ202*, *leu2Δ1*, *trp1Δ63*, *ura3-52*, *cpr7*::*KanMX*	This study
MR194	*MATa*, *kar 1–1*, *SUQ5*, *ADE2-1*, *his3Δ202*, *leu2Δ1*, *trp1Δ63*, *ura3-52*, *hsc82*::*KanMX* [*PSI* ^*+*^]	Masison’s lab
MR195	*MATa*, *kar 1–1*, *SUQ5*, *ADE2-1*, *his3Δ202*, *leu2Δ1*, *trp1Δ63*, *ura3-52*, *hsp82*::*KanMX* [*PSI* ^*+*^]	Masison’s lab
SY295	*MATa*, *kar 1–1*, *SUQ5*, *P*_*DAL5*_::*ADE2*, *his3Δ202*, *leu2Δ1*, *trp1Δ63*, *ura3-52*, *hsp82*::*KanMX* [URE3]	This study
SY297	*MATa*, *kar 1–1*, *SUQ5*, *P*_*DAL5*_::*ADE2*, *his3Δ202*, *leu2Δ1*, *trp1Δ63*, *ura3-52*, *hsc82*::*KanMX* [URE3]	This study
SY136	*MATa*, *P*_*DAL5*_::*ADE2*, *ssa1*::*Kan*, *ssa2*::*HIS3*, *ssa3*::*TRP1*, *ssa4*::*URA3-2f/pRS315P*_*SSA2*_ *-SSA2*, [URE3]	Sharma *et al*, 2011
776-6A	*MATa*, *kar 1–1*, *SUQ5*, *ADE2-1*, *his3Δ202*, *leu2Δ1*, *trp1Δ63*, *ura3-52*, [*PSI* ^*+*^]	Jung *et al*, 2001
NY02	*MATa*, *kar 1–1*, *SUQ5*, *P*_*DAL5*_::*ADE2*, *his3Δ202*, *leu2Δ1*, *trp1Δ63*, *ura3-52*, *hsc82*::*KanMX*, *hsp82*::*NatMX/ pRS316P*_*HSP82*_ *-HSP82* [URE3]	This study
NY03	*MATa*, *kar 1–1*, *SUQ5*, *P*_*DAL5*_::*ADE2*, *his3Δ202*, *leu2Δ1*, *trp1Δ63*, *ura3-52*, *hsc82*::*KanMX*, *hsp82*::*NatMX/ pRS413P*_*TEF*_ *-His*_*6*_ *-HSP82* [URE3]	This study
NY04	*MATa*, *kar 1–1*, *SUQ5*, *P*_*DAL5*_::*ADE2*, *his3Δ202*, *leu2Δ1*, *trp1Δ63*, *ura3-52*, *hsc82*::*KanMX*, *hsp82*::*NatMX/ pRS413P*_*TEF*_ *-His*_*6*_ *-HSP82(ΔMEEVD)* [URE3]	This study
NY05	*MATa*, *kar 1–1*, *SUQ5*, *P*_*DAL5*_::*ADE2*, *his3Δ202*, *leu2Δ1*, *trp1Δ63*, *ura3-52*, *hsc82*::*KanMX*, *hsp82*::*NatMX/ pRS413P*_*TEF*_ *-His*_*6*_ *-HSP82(Δ211–264)* [URE3]	This study
NY06	*MATa*, *kar 1–1*, *SUQ5*, *P*_*DAL5*_::*ADE2*, *his3Δ202*, *leu2Δ1*, *trp1Δ63*, *ura3-52*, *hsc82*::*KanMX*, *hsp82*::*NatMX/ pRS413P*_*TEF*_ *-HSP82* [URE3]	This study
NY07	*MATa*, *kar 1–1*, *SUQ5*, *P*_*DAL5*_::*ADE2*, *his3Δ202*, *leu2Δ1*, *trp1Δ63*, *ura3-52*, *hsc82*::*KanMX*, *hsp82*::*NatMX/ pRS413*_*TEF*_ *-HSP82(ΔMEEVD)* [URE3]	This study
NY14	*MATa*, *kar 1–1*, *SUQ5*, *P*_*DAL5*_::*ADE2*, *his3Δ202*, *leu2Δ1*, *trp1Δ63*, *ura3-52*, *cpr6*::*KanMX* [URE3]	This study
NY15	*MATa*, *kar 1–1*, *SUQ5*, *ADE2-1*, *his3Δ202*, *leu2Δ1*, *trp1Δ63*, *ura3-52*, *cpr7*::*KanMX* [*PSI* ^*+*^]	This study
NY16	*MATa*, *P*_*DAL5*_::*ADE2*, *ssa1*::*NatMX*, *ssa2*::*HIS3*, *ssa3*::*TRP1*, *ssa4*::*URA3-2f/pRS316P*_*SSA2*_ *-SSA2*, [URE3]	This study
NY17	*MATa*, *P*_*DAL5*_::*ADE2*, *ssa1*::*NatMX*, *ssa2*::*HIS3*, *ssa3*::*TRP1*, *ssa4*::*URA3-2f*, *cpr7*::*KanMX /pRS316P*_*SSA2*_ *-SSA2*	This study
SY269	*MATa*, *kar 1–1*, *SUQ5*, *P*_*DAL5*_::*ADE2*, *his3Δ202*, *leu2Δ1*, *trp1Δ63*, *ura3-52*, *sti1*::*KanMX* [URE3]	This study
DD168	*MATa*, *kar 1–1*, *SUQ5*, *P*_*DAL5*_::*ADE2*, *his3Δ202*, *leu2Δ1*, *trp1Δ63*, *ura3-52*, *sba1*::*KanMX* [URE3]	This study
DD169	*MATa*, *kar 1–1*, *SUQ5*, *P*_*DAL5*_::*ADE2*, *his3Δ202*, *leu2Δ1*, *trp1Δ63*, *ura3-52*, *hch1*::*KanMX* [URE3]	This study
DD170	*MATa*, *kar 1–1*, *SUQ5*, *P*_*DAL5*_::*ADE2*, *his3Δ202*, *leu2Δ1*, *trp1Δ63*, *ura3-52*, *aha1*::*KanMX* [URE3]	This study
DD171	*MATa*, *kar 1–1*, *SUQ5*, *P*_*DAL5*_::*ADE2*, *his3Δ202*, *leu2Δ1*, *trp1Δ63*, *ura3-52*, *ppt1*::*KanMX* [URE3]	This study
DD172	*MATa*, *kar 1–1*, *SUQ5*, *P*_*DAL5*_::*ADE2*, *his3Δ202*, *leu2Δ1*, *trp1Δ63*, *ura3-52*, *tah1*::*KanMX* [URE3]	This study
AY3	*MATa*, *kar 1–1*, *SUQ5*, *ADE2-1*, *his3Δ202*, *leu2Δ1*, *trp1Δ63*, *ura3-52*, *hch1*::*KanMX* [*PSI* ^*+*^]	This study
AY4	*MATa*, *kar 1–1*, *SUQ5*, *ADE2-1*, *his3Δ202*, *leu2Δ1*, *trp1Δ63*, *ura3-52*, *aha1*::*KanMX* [*PSI* ^*+*^]	This study
AY5	*MATa*, *kar 1–1*, *SUQ5*, *ADE2-1*, *his3Δ202*, *leu2Δ1*, *trp1Δ63*, *ura3-52*, *cpr6*::*KanMX* [*PSI* ^*+*^]	This study
AY6	*MATa*, *kar 1–1*, *SUQ5*, *ADE2-1*, *his3Δ202*, *leu2Δ1*, *trp1Δ63*, *ura3-52*, *sba1*::*KanMX* [*PSI* ^*+*^]	This study
AY7	*MATa*, *kar 1–1*, *SUQ5*, *ADE2-1*, *his3Δ202*, *leu2Δ1*, *trp1Δ63*, *ura3-52*, *ppt1*::*KanMX* [*PSI* ^*+*^]	This study
AY8	*MATa*, *kar 1–1*, *SUQ5*, *ADE2-1*, *his3Δ202*, *leu2Δ1*, *trp1Δ63*, *ura3-52*, *sti1*::*KanMX* [*PSI* ^*+*^]	This study
AY9	*MATa*, *kar 1–1*, *SUQ5*, *ADE2-1*, *his3Δ202*, *leu2Δ1*, *trp1Δ63*, *ura3-52*, *tah1*::*KanMX* [*PSI* ^*+*^]	This study
1566	*kar 1–1*, *SUQ5*, *ADE2-1*, *his3Δ202*, *leu2Δ1*, *trp1Δ63*, *ura3-52*, [*PSI* ^*+*^]^Sc4^	Masison’s lab
1567	*kar 1–1*, *SUQ5*, *ADE2-1*, *his3Δ202*, *leu2Δ1*, *trp1Δ63*, *ura3-52*, [*PSI* ^*+*^]^Sc37^	Masison’s lab
AY11	*kar 1–1*, *SUQ5*, *ADE2-1*, *his3Δ202*, *leu2Δ1*, *trp1Δ63*, *ura3-52*, *cpr7*::*KanMX*, ([*PSI* ^*+*^]^Sc4^)	This study
AY12	*kar 1–1*, *SUQ5*, *ADE2-1*, *his3Δ202*, *leu2Δ1*, *trp1Δ63*, *ura3-52 cpr7*::*KanMX*, ([*PSI* ^*+*^]^Sc37^)	This study

**Table 2:** 

Plasmid	Marker	Reference
pRS413P_TEF_-CPR7	HIS3	This study
pRS413P_TEF_-CPR6	HIS3	This study
pRS413P_TEF_-6PPI/7TPR	HIS3	This study
pRS413P_TEF_-7PPI/6TPR	HIS3	This study
pRS413P_TEF_-7TPR	HIS3	This study
pRS316P_CPR7_-CPR7	URA3	This study
pRS316P_CPR6_-CPR6	URA3	This study
pRS426P_CNS1_-CNS1	URA3	This study
pKT41-URE2	Ampicillin	Sharma *et al*, 2011
pET29bHTV-CPR6	Kanamycin	This study
pET29bHTV-CPR7	Kanamycin	This study
pET29bHTV-7TPR	Kanamycin	This study
pRS426P_GPD_-His_6_-HSP82	URA3	This study
pRS426P_GPD_-His_6_-HSC82	URA3	This study
pRS413P_TEF_-His_6_-HSP82	HIS3	This study
pRS413P_TEF_-HSP82	HIS3	This study
pRS413P_TEF_-His_6_-HSP82ΔMEEVD	HIS3	This study
pRS413P_TEF_-HSP82ΔMEEVD	HIS3	This study
pRS413P_TEF_-His_6_-HSP82Δ211–264	HIS3	This study
pRS316P_HSP82_-HSP82	URA3	This study
pRS315P_SSA2_-SSA1	LEU2	Sharma & Masison, 2008
pRS315P_SSA2_-SSA2	LEU2	Sharma & Masison, 2008
pRS315P_SSA2_-SSA3	LEU2	Sharma & Masison, 2008
pRS315P_SSA2_-SSA4	LEU2	Sharma & Masison, 2008
pRS316P_GAL1_-FLAG-vSrc	URA3	This study
pRS426P_GPD_-GFP	URA3	This study
pRS426P_GPD_-URE2-GFP	URA3	This study
pRS413P_TEF_-His_6_-CPR7	HIS3	This study

The genes encoding Cpr6 or Cpr7 were cloned under their respective native promoters in pRS316 to construct pRS316P_CPR6_-CPR6 or pRS316P_CPR7_-CPR7, respectively. Similarly, pRS413P_TEF_-CPR6 and pRS413P_TEF_-CPR7 were constructed to express Cpr6 or Cpr7 respectively from the *TEF* promoter. Overlap PCR was carried out to construct genes encoding hybrid proteins 6PPI/7TPR or 7PPI/6TPR. The *6PPI/7TPR* encodes amino acids (a.a.) 1–176 of Cpr6 and 198–393 of Cpr7. Similarly *7PPI*/*6TPR* encodes a.a. 1–197 of Cpr7 and 177–371 of Cpr6. The full length PCR amplified product was further digested with BamHI and XhoI, and subcloned into plasmid pRS413P_TEF_. Similarly plasmid pRS413P_TEF_-7TPR, encoding the TPR domain of Cpr7 (a.a. 193–393)(henceforth 7TPR), was constructed by restriction digestion (BamHI and XhoI) followed by ligation of a PCR amplified gene product encoding the TPR domain of Cpr7 into plasmid pRS413P_TEF_ digested with similar restriction enzymes. For immunoblot and protein purification of Cpr7 and Cpr6, a short tract of Hexa-His-tag was added at the 5’ end of gene encoding Cpr7 or Cpr6 using standard PCR-based recombinant technology. For expression in *E*.*coli*, pET29bHTV-CPR6, pET29bHTV-CPR7 or pET29bHTV-7TPR was constructed to encode from 5’ to 3’ direction, a Hexa-His tag, and a TEV protease recognition site followed by the gene encoding CPR6, CPR7 or 7TPR, respectively.

### Media and growth conditions

Media and growth conditions are as described previously [64]. ½ YPD medium contains 0.5% yeast extract, 2% peptone, 2% dextrose and 2% agar. Liquid YPAD is similar to ½ YPD except it contains 1% yeast extract and 200mg/L adenine. SD and SGal are synthetic dextrose and synthetic galactose minimal media, respectively. Cells were grown at 30°C unless otherwise stated.

### Diploid formation and tetrad analysis

The heterologous diploid (*CPR7/cpr7*::*KanMX*) was constructed by mating SY187 [URE3] *MATα* with NY01 *MATa* strain (*cpr7Δ*[ure-o]) and selecting on adenine deficient medium containing G418 (200 μg/ml). Diploids were further streaked to pure colonies on ½ YPD plates and further confirmed by replica plating onto adenine deficient SD plate containing G418. Diploids were sporulated on a 2% potassium acetate plate and tetrad dissection was carried out on ½ YPD.

### Monitoring [URE3] and [*PSI* ^*+*^]

Gln3 is an activator of *DAL5* promoter. When cells are grown in the presence of a good nitrogen source such as standard ammonium containing media, Ure2 binds to transcription factor Gln3 and represses the *DAL5* promoter. To monitor [URE3], our strains encode *ADE2* controlled by the *DAL5* promoter. In [ure-o] (lacking [URE3]) cells, functional Ure2 represses transcription of *ADE2* so cells do not grow in the absence of adenine and remain red on limiting adenine media. [URE3] cells contain predominantly inactive Ure2, which relieves repression of *DAL5* and allows expression of Ade2. Thus [URE3] cells grow on media lacking adenine and remain white on limiting adenine media.

Sup35 is a translation termination factor and thus its conversion to the functionally inactive [*PSI* ^*+*^] form leads to suppression of *ade2-1* nonsense allele. This suppression also requires the weak tRNA suppressor Sup16 (encoded by *SUQ5*), which alone does not suppress *ade2-1*. Thus, in cells propagating [*PSI* ^*+*^], enhanced nonsense suppression of *ade2-1* leads to the synthesis of Ade2. In contrast, [*psi* ^-^] cells lack functional Ade2 and thus are unable to grow in the absence of externally added adenine and remain red when grown on limiting adenine.

The prion variants of [URE3] and strong [*PSI* ^*+*^] in our strains are of unknown origin. Our strains 1566 and 1567 are kind gift from Dr. Daniel Masision, and contain the [*PSI* ^*+*^]^Sc4^ and [*PSI* ^*+*^]^Sc37^ variants respectively that were introduced by cytoduction from J. Weissman strains JW127 and JW129 [51].

### Protein purification

For Cpr7 purification, the plasmid pET29bHTV-CPR7 was transformed into *Escherichia coli* strain Rosetta 2(DE3) (Invitrogen). The pre-grown culture of optical density at 600nm (O.D._600nm_) ~0.6 was induced with isopropyl-β-D-thiogalactopyranoside (IPTG) at 18°C. Cells were lysed and the protein was purified from supernatant using cobalt based Talon metal affinity resin. Cpr7 was eluted with 300mM imidazole and further incubated overnight with His_6_-TEV (molar ratio, Cpr7/His-TEV:20/1) protease at 4°C. The sample was extensively dialyzed and further incubated with cobalt metal affinity column and the His_6_-tag cleaved Cpr7 protein was collected as unbound fraction. The protein was further purified using anion exchange (Mini Q) chromatography. Protein purity was verified using SDS-PAGE and identity was confirmed using Mass spectrometry. Cpr6 and 7TPR were purified using the procedure described above for Cpr7.

The plasmid pKT41-Ure2 (kind gift from Dr. Reed Wickner, National Institute of Health, Bethesda) encodes Ure2 with an N-terminal Hexa-His-tag. The plasmid was transformed into *E*.*coli* strain Rosetta 2(DE3) and Ure2p was purified using cobalt based Talon metal affinity resin as described before [52].

### Ure2 fibrillation assay

Thioflavin T (ThT) (500μM) was added to 48 μM of purified Ure2 with and without Cpr6, Cpr7 or 7TPR (30 μM each) in a 96 well plate. The plate was incubated at 37°C with a shaking speed of 900 rpm in linear mode in a multimode plate reader (TECAN infinite M200 PRO). Fluorescence kinetics was measured after every 15 minutes with emission wavelength of 485 nm upon excitation at 450 nm. Each experiment was repeated at least three times.

### Western analysis

Cells were lysed using lysis buffer (phosphate-buffered saline with 0.2% TritonX-100 and protease inhibitor cocktail) by vortexing with glass beads and lysate was fractionated into supernatant and pellet. About 10 μg of total protein was separated on sodium dodecyl sulfate polyacrylamide gels and transferred onto polyvinylidene fluoride membranes. Anti His_6_-tag antibodies (Pierce Biotechnology) were used to capture His_6_-Cpr7. For Pull-down assay, purified His_6_-Cpr7 was bound over cobalt based metal affinity resin and yeast lysate was passed through the resin. The resin was washed and the bound fractions were probed with desired antibodies.

## Supporting Information

S1 Fig Hsp90 inhibition does not affect Prion propagation. (A) SY136 strain harbouring plasmid encoding FLAG-v-Src under galactose inducible promoter was grown in liquid SD medium lacking uracil from O.D._600nm_ of 0.02 to 1.0. Expression of the Hsp90 client FLAG-v-Src was induced by shifting cells (O.D._600nm_ 0.5) to SGal medium containing DMSO or 50μM 17-AAG. Cells were lysed and lysate was immunoblotted using antibodies against Hsp70 and FLAG-tag. As seen, incubation with 17-AAG results in increased Hsp70 level and decreased v-Src level. (B) Cells were grown in the presence and absence of 17-AAG as described above. Cells were harvested and total RNA was isolated using HiPurA Yeast RNA Purification Kit (MB611) following manufacturer’s protocol. The cDNA was made using cDNA synthesis kit (Verso from Thermo Scientific, AB1453B) and used as template for real time qRT-PCR. As shown, no significant difference of mRNA was observed in cells treated with 17-AAG compared to those incubated with DMSO. (C) *Hsc82Δ* [URE3] cells were grown overnight and further subcultured in YPAD liquid medium at O.D._600nm_ from 0.2 to 1.7 in the presence of DMSO or 100μM 17-AAG. The cells were re-subcultured under similar conditions and spread onto ½ YPD medium. As seen by white colony color phenotype, no effect on [URE3] stability was observed upon incubation with 17-AAG.
(TIF)

S2 Fig Strains harbouring Hsp90 variants have similar expression of major heat shock proteins. About 10μg of the yeast lysate proteins from the indicated strains were loaded per lane and immunoblotted with antibodies directed against Hsp70, Ydj1, Hsp90 and Sse1. As seen, these Hsp proteins are expressed at similar level.
(TIF)

S3 Fig Cpr7 deletion has no significant effect on strong or weak variant of [*PSI* ^*+*^] prions. The gene encoding Cpr7 was deleted in strains harboring strong (1566) or weak (1567) [*PSI* ^*+*^] variants. **(A)** The indicated strains were streaked onto a ½ YPD plate and grown for 2 days at 30°C and 1 days at room temperature. (B) The strains were patched onto ½ YPD and grown for 1 day at 30°C. The plate was further replicated onto solid ½ YPD or SD medium lacking adenine and grown for 4 days at 30°C.
(TIF)

S4 Fig Cpr7 deletion does not affect adenine biosynthesis pathway. Wild type [URE3], wt [ure-o] or *cpr7Δ* strain were transformed with pRS316P_CPR7_-CPR7 or empty plasmid pRS316. About 5–6 transformants were pooled into liquid medium and further grown from O.D._600nm_ of 0.02 to 1.7, and plated onto uracil deficient solid medium with limiting adenine. As shown, *cpr7Δ* strain transformed with *CPR7* plasmid remains red suggesting that the appearance of red colony color phenotype in *cpr7Δ* strain is due to loss of [URE3].
(TIF)

S5 Fig **(A)** The NY17 strain was constructed by deleting gene encoding Cpr7 in NY16 strain expressing Ssa2 as sole Ssa Hsp70 source regulated by the Ssa2 promoter. The indicated strains were streaked onto a ½ YPD plate and incubated at 30°C for 2 days. Cells were then replicated onto solid SD medium lacking adenine. As seen, Cpr7 deletion results in loss of [URE3] prion in NY17 strain **(B)** About 10μg of the yeast lysate proteins from indicated strain was loaded per lane and Hsp70 level was measured using anti Hsp70 antibodies similar to as described above in Fig 3B. Lower panel shows the same blot stained with amido-black as loading and transfer control. **(C)** NY16 [URE3] or [ure-o] strain was transformed with either empty plasmid (ev) or that encoding Ssa2 under Ssa2 promoter. As seen, the presence of additional copies of gene expressing Ssa2 supports [URE3] stability.
(TIF)

S6 Fig Cpr7 interacts Ure2 in vivo. SY187 [ure-o] was co-transformed with pRS413P_TEF_-His_6_-Cpr7 & pRS426P_GPD_-Ure2-GFP or pRS426P_GPD_-GFP. Cells were grown from O.D._600nm_ of 0.1 to 1. Cells were lysed using a glass beads and 5mg of lysate proteins were loaded onto cobalt metal affinity resin at 4°C for 2 hrs. Upon washing, bound proteins were eluted using 25mM EDTA and probed with anti-GFP or anti-His_6_ antibody.
(TIF)

S7 Fig Cpr7 alone does not affect ThT fluorescence emission. In vitro Ure2 fibrillation was monitored using ThT assay as described in Fig 7 (**A**) Reactions containing Cpr7 showed a substantial increase in ThT fluorescence intensity, which saturated at about 200 min. (**B**) After allowing the reaction to continue for about 12 hours at 37°C and 24 hours at 4°C the mixture was fractionated into supernatant and pellet. Protein concentration in supernatant of reactions with Ure2 and Ure2 plus Cpr7 was found to be 0.87μM and 1.14 μM respectively (in contrast to 48μM Ure2 and 30μM Cpr7 at the beginning of the reaction). The pellet was resuspended in an equal volume (200μl) of 25mM HEPES, 150mM NaCl and ThT fluorescence was read at 485nm upon excitation at 450nm. As seen, similar increase in fluorescence intensity was observed with pellet from Ure2 or Ure2 with Cpr7 suggesting that Cpr7 by itself has no effect on ThT fluorescence.
(TIF)
