# Supplementary figures and images for: Hsp90-Associated Immunophilin Homolog Cpr7 Is Required for the Mitotic Stability of [URE3] Prion in Saccharomyces cerevisiae
Source: PLoS Genet. 2015 Oct 16;11(10):e1005567. doi: 10.1371/journal.pgen.1005567 (PMC4608684; doi:10.1371/journal.pgen.1005567)

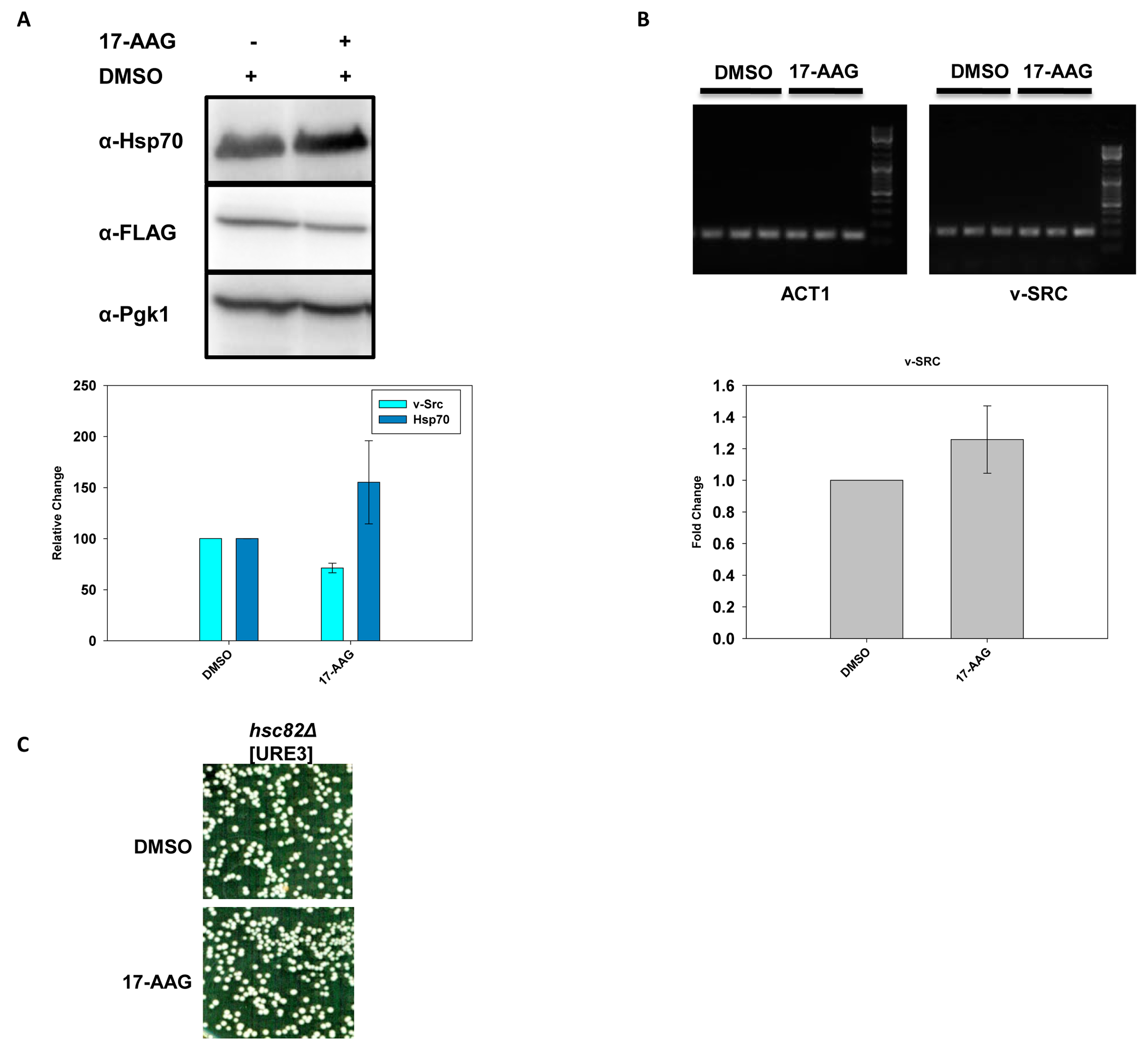

Supplement: S1 Fig — (A) SY136 strain harbouring plasmid encoding FLAG-v-Src under galactose inducible promoter was grown in liquid SD medium lacking uracil from O.D.600nm of 0.02 to 1.0. Expression of the Hsp90 client FLAG-v-Src was induced by shifting cells (O.D.600nm 0.5) to SGal medium containing DMSO or 50μM 17-AAG. Cells were lysed and lysate was immunoblotted using antibodies against Hsp70 and FLAG-tag. As seen, incubation with 17-AAG results in increased Hsp70 level and decreased v-Src level. (B) Cells were grown in the presence and absence of 17-AAG as described above. Cells were harvested and total RNA was isolated using HiPurA Yeast RNA Purification Kit (MB611) following manufacturer’s protocol. The cDNA was made using cDNA synthesis kit (Verso from Thermo Scientific, AB1453B) and used as template for real time qRT-PCR. As shown, no significant difference of mRNA was observed in cells treated with 17-AAG compared to those incubated with DMSO. (C) Hsc82Δ [URE3] cells were grown overnight and further subcultured in YPAD liquid medium at O.D.600nm from 0.2 to 1.7 in the presence of DMSO or 100μM 17-AAG. The cells were re-subcultured under similar conditions and spread onto ½ YPD medium. As seen by white colony color phenotype, no effect on [URE3] stability was observed upon incubation with 17-AAG. (TIF) [file pgen.1005567.s001.tif]

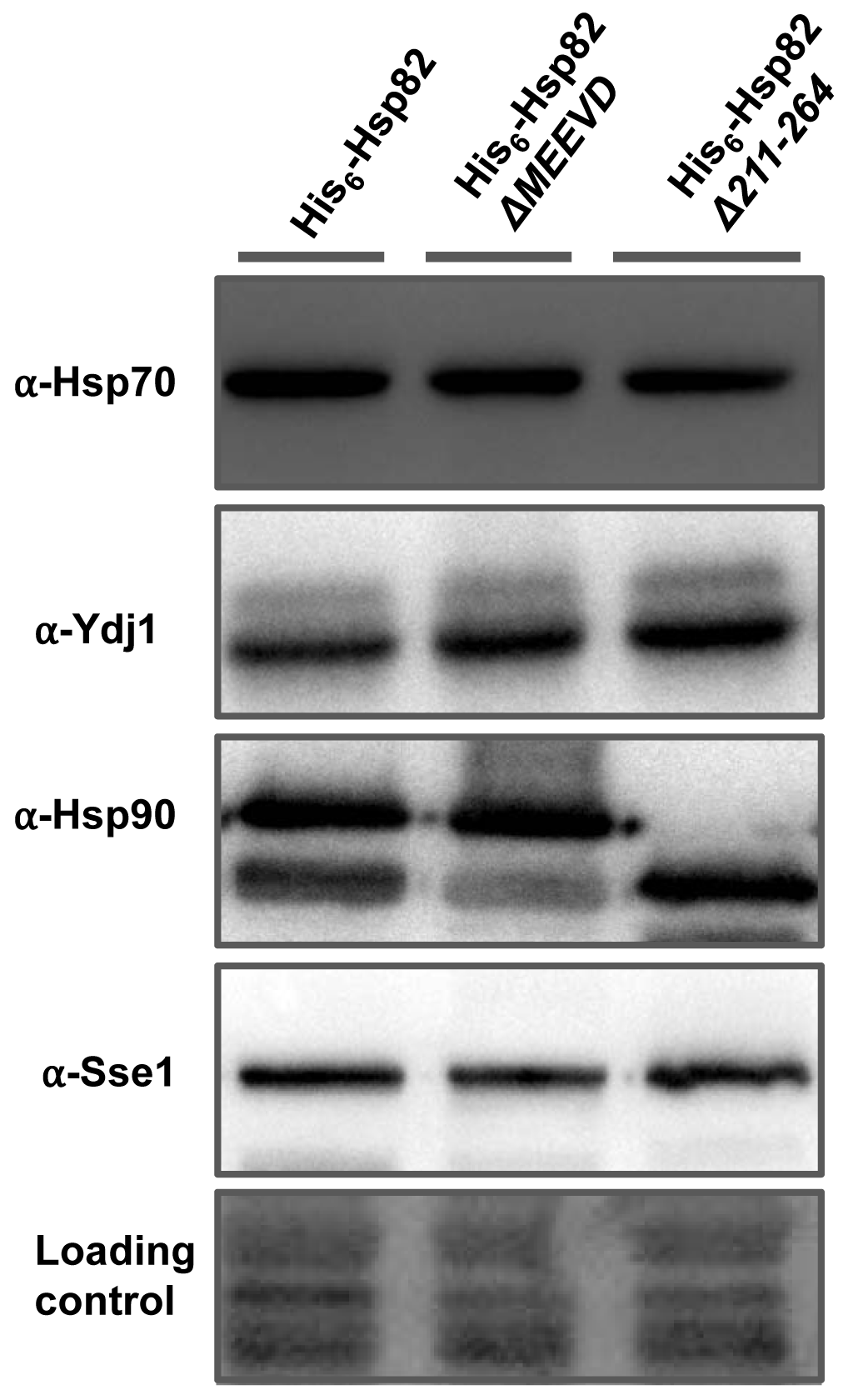

Supplement: S2 Fig — About 10μg of the yeast lysate proteins from the indicated strains were loaded per lane and immunoblotted with antibodies directed against Hsp70, Ydj1, Hsp90 and Sse1. As seen, these Hsp proteins are expressed at similar level. (TIF) [file pgen.1005567.s002.tif]

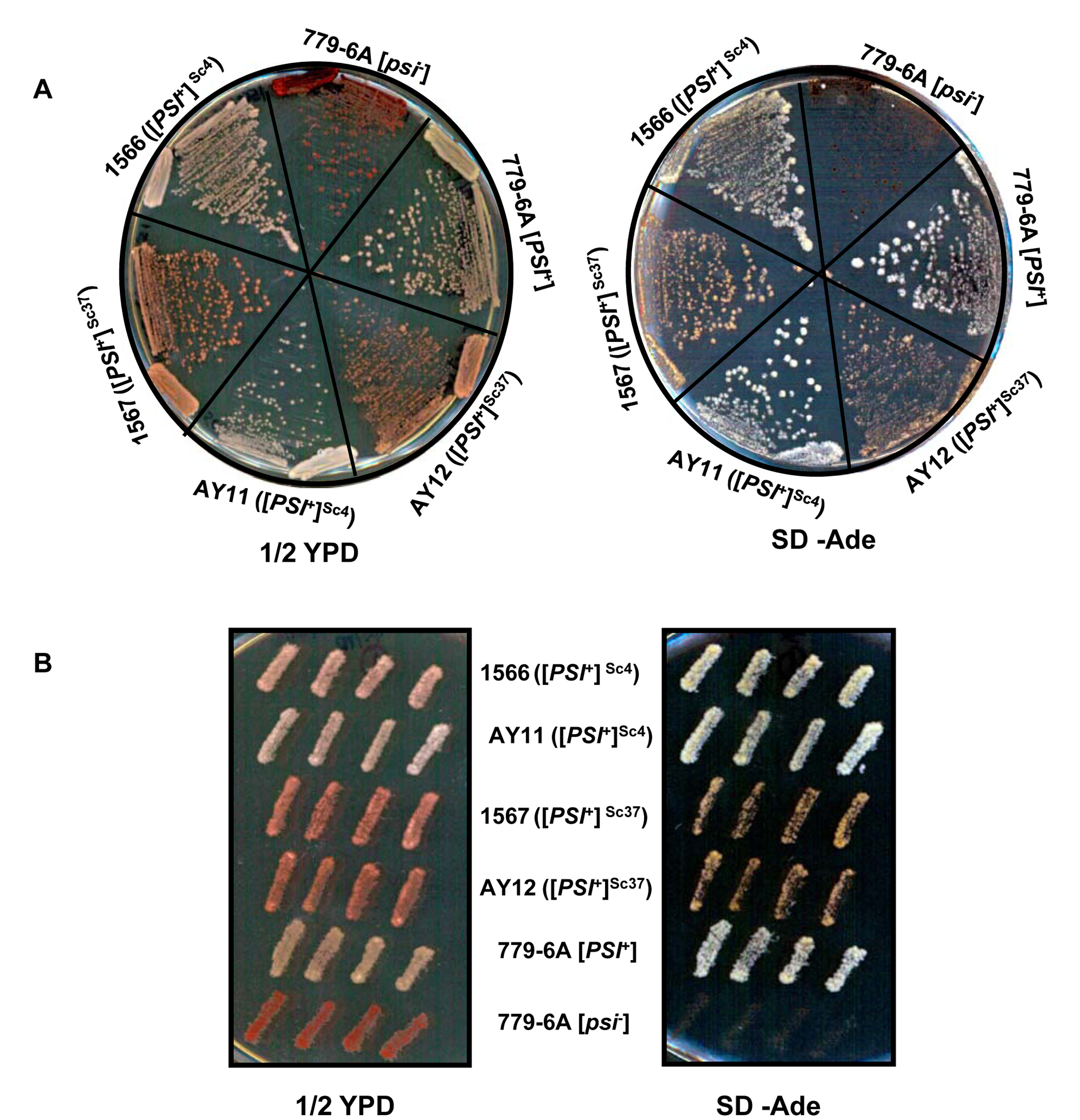

Supplement: S3 Fig — The gene encoding Cpr7 was deleted in strains harboring strong (1566) or weak (1567) [PSI +] variants. (A) The indicated strains were streaked onto a ½ YPD plate and grown for 2 days at 30°C and 1 days at room temperature. (B) The strains were patched onto ½ YPD and grown for 1 day at 30°C. The plate was further replicated onto solid ½ YPD or SD medium lacking adenine and grown for 4 days at 30°C. (TIF) [file pgen.1005567.s003.tif]

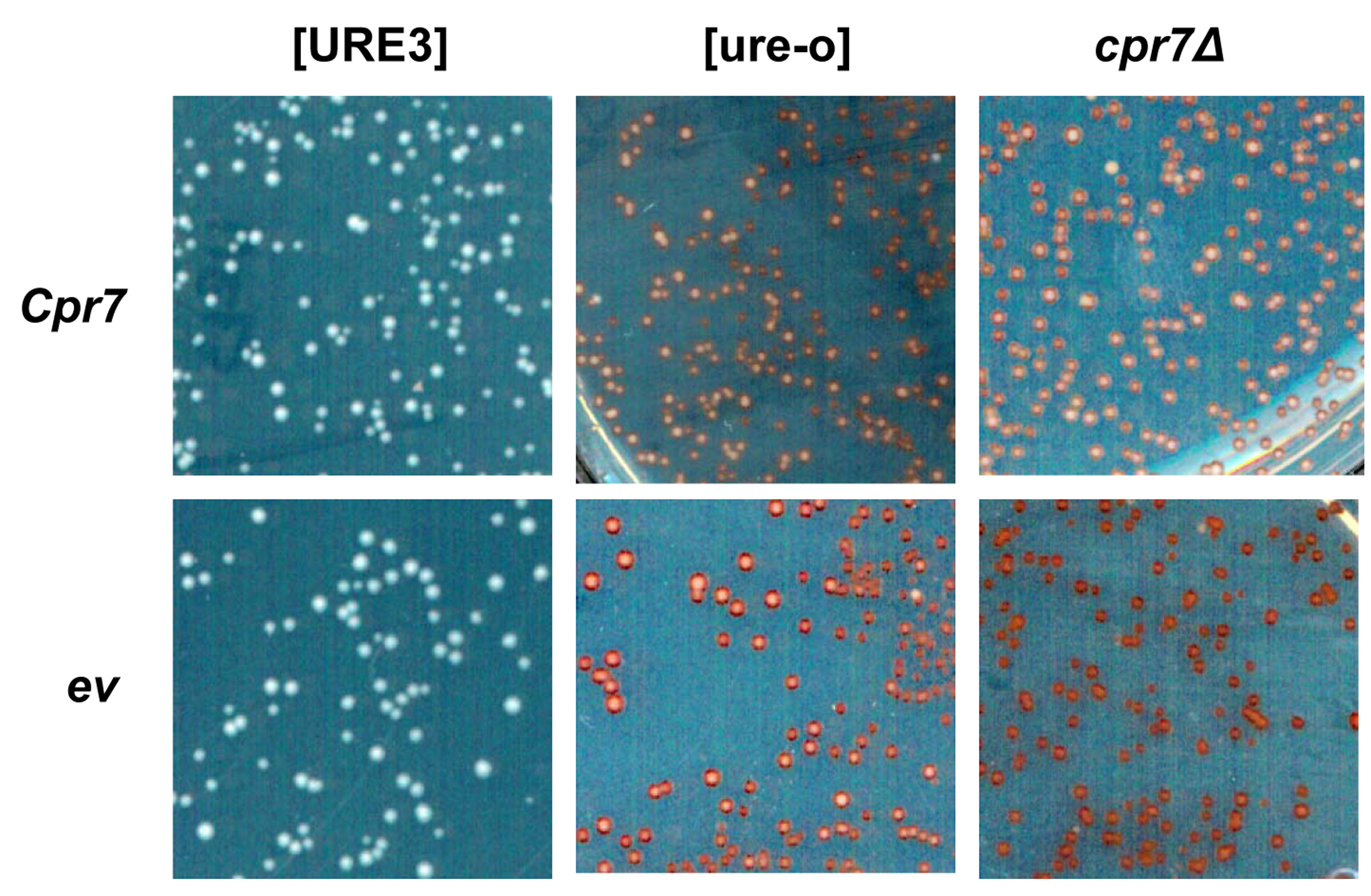

Supplement: S4 Fig — Wild type [URE3], wt [ure-o] or cpr7Δ strain were transformed with pRS316PCPR7-CPR7 or empty plasmid pRS316. About 5–6 transformants were pooled into liquid medium and further grown from O.D.600nm of 0.02 to 1.7, and plated onto uracil deficient solid medium with limiting adenine. As shown, cpr7Δ strain transformed with CPR7 plasmid remains red suggesting that the appearance of red colony color phenotype in cpr7Δ strain is due to loss of [URE3]. (TIF) [file pgen.1005567.s004.tif]

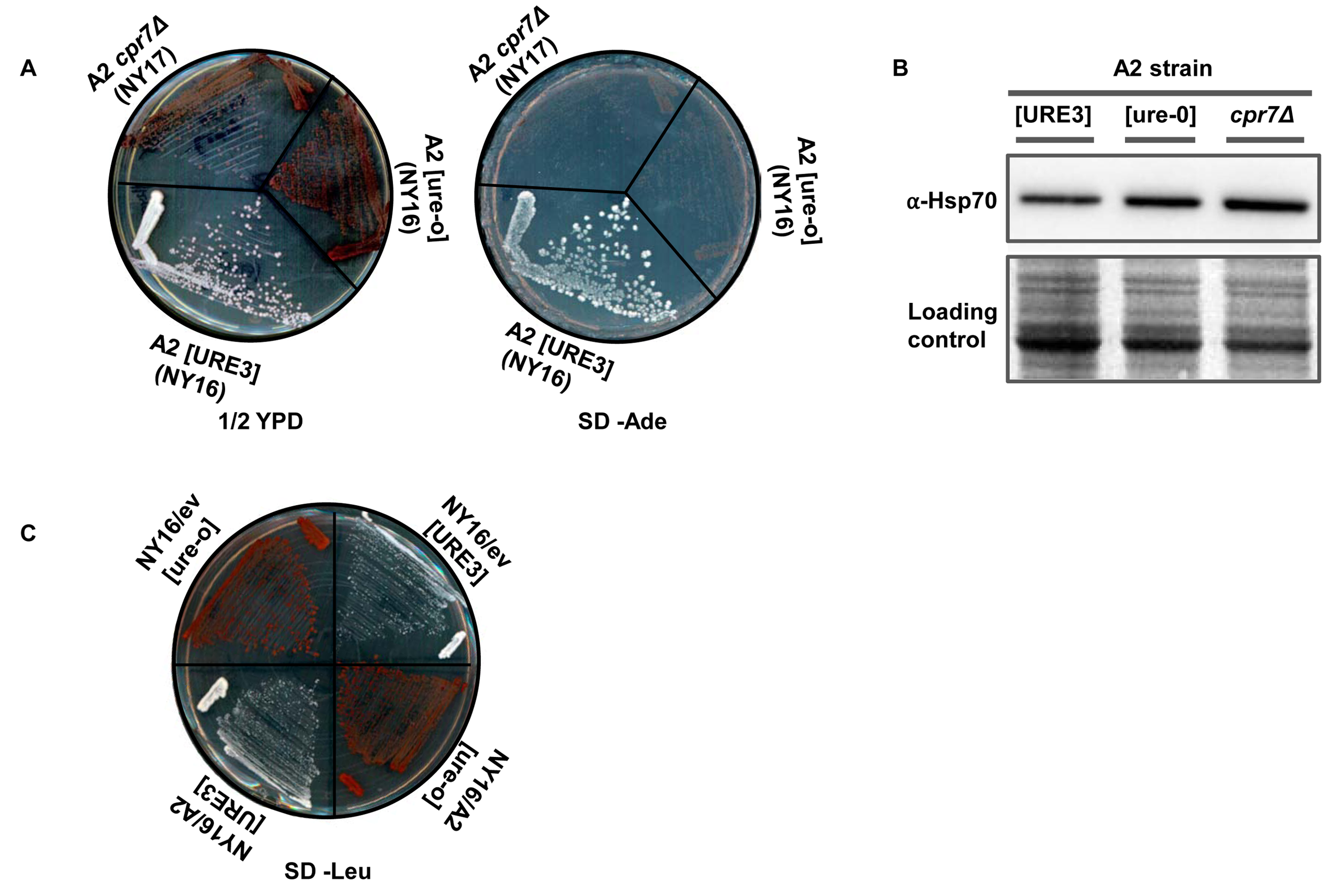

Supplement: S5 Fig — (A) The NY17 strain was constructed by deleting gene encoding Cpr7 in NY16 strain expressing Ssa2 as sole Ssa Hsp70 source regulated by the Ssa2 promoter. The indicated strains were streaked onto a ½ YPD plate and incubated at 30°C for 2 days. Cells were then replicated onto solid SD medium lacking adenine. As seen, Cpr7 deletion results in loss of [URE3] prion in NY17 strain (B) About 10μg of the yeast lysate proteins from indicated strain was loaded per lane and Hsp70 level was measured using anti Hsp70 antibodies similar to as described above in Fig 3B. Lower panel shows the same blot stained with amido-black as loading and transfer control. (C) NY16 [URE3] or [ure-o] strain was transformed with either empty plasmid (ev) or that encoding Ssa2 under Ssa2 promoter. As seen, the presence of additional copies of gene expressing Ssa2 supports [URE3] stability. (TIF) [file pgen.1005567.s005.tif]

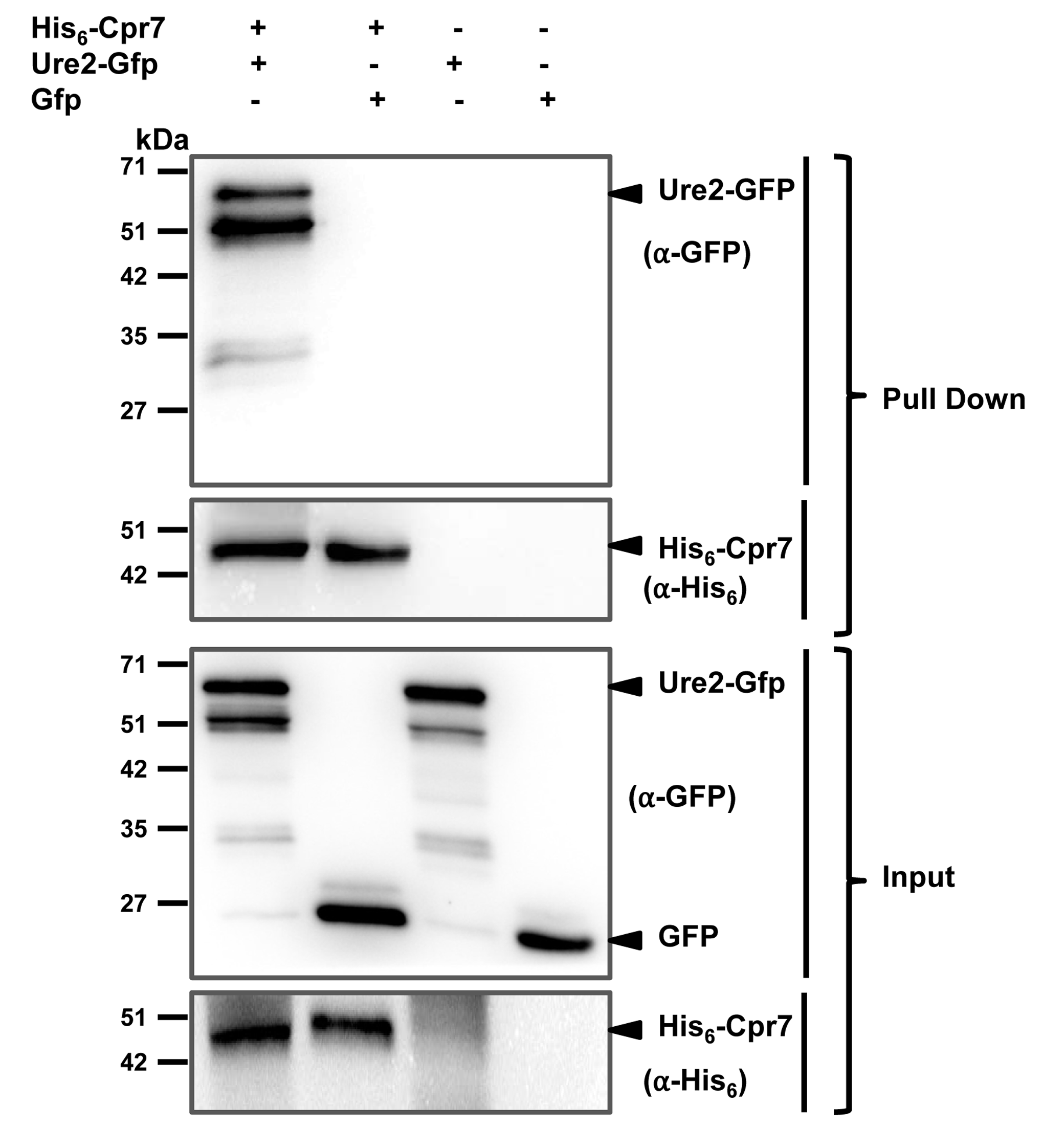

Supplement: S6 Fig — SY187 [ure-o] was co-transformed with pRS413PTEF-His6-Cpr7 & pRS426PGPD-Ure2-GFP or pRS426PGPD-GFP. Cells were grown from O.D.600nm of 0.1 to 1. Cells were lysed using a glass beads and 5mg of lysate proteins were loaded onto cobalt metal affinity resin at 4°C for 2 hrs. Upon washing, bound proteins were eluted using 25mM EDTA and probed with anti-GFP or anti-His6 antibody. (TIF) [file pgen.1005567.s006.tif]

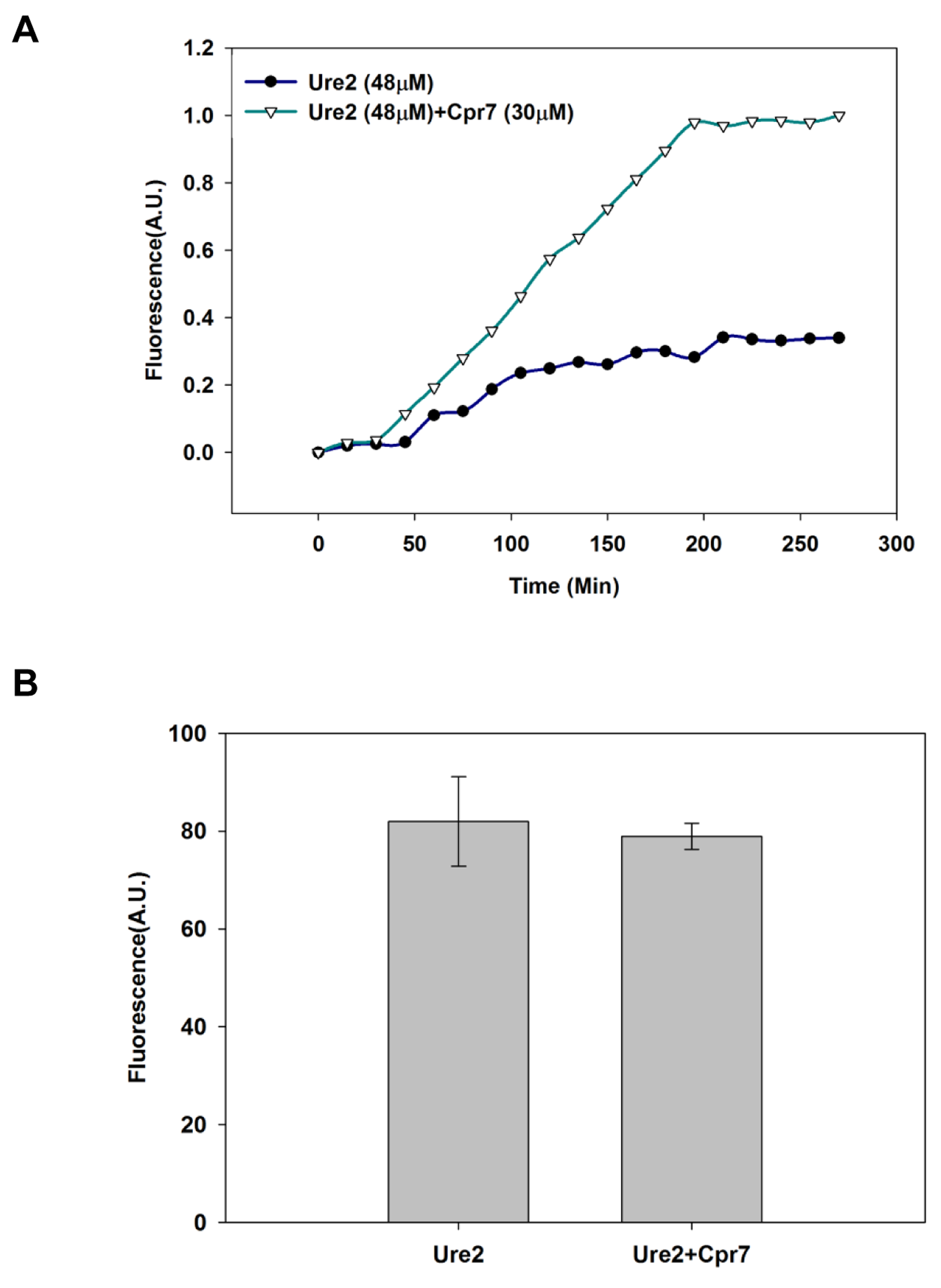

Supplement: S7 Fig — In vitro Ure2 fibrillation was monitored using ThT assay as described in Fig 7 (A) Reactions containing Cpr7 showed a substantial increase in ThT fluorescence intensity, which saturated at about 200 min. (B) After allowing the reaction to continue for about 12 hours at 37°C and 24 hours at 4°C the mixture was fractionated into supernatant and pellet. Protein concentration in supernatant of reactions with Ure2 and Ure2 plus Cpr7 was found to be 0.87μM and 1.14 μM respectively (in contrast to 48μM Ure2 and 30μM Cpr7 at the beginning of the reaction). The pellet was resuspended in an equal volume (200μl) of 25mM HEPES, 150mM NaCl and ThT fluorescence was read at 485nm upon excitation at 450nm. As seen, similar increase in fluorescence intensity was observed with pellet from Ure2 or Ure2 with Cpr7 suggesting that Cpr7 by itself has no effect on ThT fluorescence. (TIF) [file pgen.1005567.s007.tif]
